# Integrative emotion regulation relates to sympathy and support for outgroups—Independent of situational outgroup behaviour

**DOI:** 10.1371/journal.pone.0296520

**Published:** 2024-01-05

**Authors:** Lara Ditrich, Jonas Reinhardt, Guy Roth, Kai Sassenberg

**Affiliations:** 1 Social Processes Lab, Leibniz-Institut für Wissensmedien (IWM), Tübingen, Germany; 2 Department of Education, Ben-Gurion University of the Negev, Beer Sheva, Israel; 3 Department of Psychology, University of Tübingen, Tübingen, Germany; Queen Mary University of London School of Business and Management, UNITED KINGDOM

## Abstract

Intergroup emotions powerfully shape intergroup relations. Anger and fear fuel, while hope and sympathy reduce intergroup strife. This implies that emotion regulation may play an important role in improving intergroup relations. Broadening the scope of prior research, we herein investigate the potential benefits of integrative emotion regulation for improving intergroup relations. Integrative emotion regulation involves actively paying attention to emotions to determine which information they provide. Interindividual differences in the use of integrative emotion regulation correlate with sympathy and supportiveness towards outgroups, but why this is the case is unclear. We tested two possible explanations: a person effect (i.e., interindividual differences in integrative emotion regulation shape respondents’ general outlook on outgroups) and a person-situation interaction effect (i.e., integrative emotion regulation reduces the impact of situational factors that would typically dampen sympathy, thereby shaping situation-specific responses to outgroups). In four experiments (total *N* = 984), we manipulated outgroup behaviour and measured interindividual differences in integrative emotion regulation. We found no interaction between integrative emotion regulation and outgroup behaviour in predicting outgroup-directed sympathy and supportiveness. Instead, integrative emotion regulation consistently correlated positively with supportiveness, mediated by sympathy. These findings suggest that those high in integrative emotion regulation have a more positive, general outlook on outgroups than those low in integrative emotion regulation, but being high in integrative emotion does not alter situational responses.

## Introduction

Intergroup relations–between young and older persons, immigrants and local residents, Israelis and Palestinians–are often emotionally charged and fraught with conflict. Given the pervasive impact of emotions on intergroup relations, specific forms of emotion regulation might prove instrumental in promoting positive intergroup relations. To date, researchers have mainly focused on strategies that reduce negative emotions towards outgroup members [1,2]. At the same time, less attention has been devoted to emotion regulation strategies that might enhance sympathy for outgroup members. Given that sympathy is a key precursor of prosocial behaviour [3] and plays an important role in shaping intergroup relations [4–6], we herein address this astonishing research gap. Specifically, we focus on an emotion regulation strategy that has previously been shown to correlate with sympathy and supportiveness [4,5], but has itself received limited attention in intergroup contexts: integrative emotion regulation (IER; [6,7]). IER involves (a) actively exploring one’s emotions and (b) integrating the information these provide with other aspects of the self. While initial results suggest that interindividual differences in IER correlate positively with sympathy and support towards others, including outgroup members [4,5], the source of this correlation is unclear. We seek to close this gap by proposing and testing two possible explanations for the IER-sympathy-supportiveness link.

On the one hand, this link could be due to interindividual differences in IER promoting an outlook on outgroups characterised by sympathy and supportiveness that transcends different situations. In what follows, we refer to this explanation as the "person hypothesis". On the other hand, the IER-sympathy link could also be due to interindividual differences in IER affecting how individuals deal with situational factors that would typically be non-conducive to the experience of sympathy. Below, we call this explanation the "person-situation interaction hypothesis". Pitting these two hypotheses against each other makes three contributions to the literature. It improves our understanding of how and why IER might contribute to improved intergroup relations. Thereby, it sheds light on the nature of IER effects and broadens the scope of emotion regulation strategies considered as potential antidotes to continued intergroup strife. Moreover, it allows us to test whether the positive relation between IER and sympathy and (consequently) supportiveness towards an outgroup documented in prior work [4] replicates across several outgroups.

### Emotions and emotion regulation in intergroup conflicts

Emotions substantially shape interactions between members of different social groups. Anger, hatred, or fear are associated with negative tendencies like reduced forgiveness (e.g., [8]; for a meta-analysis, see [9]) and enhanced support for violent military action (e.g., [10,11]). Moreover, they undermine empathy, compromise, and negotiations [8]. In contrast, emotions like hope and sympathy appear to be beneficial for intergroup relations.

Hope is linked to the perception that things may change for the better in the future and fosters support for conciliatory policies [12]. Sympathy (sometimes also called empathy or empathic concern; e.g. [13]), in turn, describes "other-oriented feelings congruent with the perceived welfare of another individual" [14, p. 621]. Its antecedents include perspective-taking and a common ingroup identity (for a summary, see [15]). Making it a particularly intriguing variable in an intergroup context, several studies demonstrate its relevance for intergroup relations (for reviews, see [16–18]). Moreover, experimental studies demonstrate that individuals instructed to sympathise with others display greater supportiveness vis-à-vis the target and the target’s ingroup (e.g., [13,19,20]; for a review, see [3]).

Owing to this important role emotions play in shaping intergroup conflicts, the last 15 years have seen a steady increase in research investigating whether emotion regulation strategies can be used to foster positive intergroup relations. Most widely studied in this regard are different forms of reappraisal, a strategy that aims at changing a situation’s cognitive meaning in a way that alters its emotional impact [21]. Thus far, the results are promising: participants reported less anger and other negative emotions towards the outgroup, less support for aggressive policies, less political intolerance, but more hope and support for conciliatory, peaceful policies [1,2,22]. Thus, fostering reappraisal appears to be a fruitful way of promoting intergroup reconciliation by reducing negative intergroup emotions–but not necessarily by fostering sympathetic responses [2]. Unfortunately, the narrow focus on reappraisal in prior work means that we, to date, know little about whether and if so why other forms of emotion regulation might have similar reconciliation-enhancing effects. We herein take a step towards closing these research gaps by focusing on sympathy as a potentially reconciliation-enhancing response and addressing a different form of emotion regulation: integrative emotion regulation.

### Integrative emotion regulation–linked to sympathy and supportiveness

Integrative emotion regulation reflects deliberately using emotional experiences as informational inputs [7] and involves two closely interrelated aspects: (a) a non-defensive awareness of one’s emotions which, in turn, allows (b) actively taking an interest in and integrating them with other aspects of the self, such as goals and values [23]. The information gained through this process then guides adaptive behaviour [7]. Thus, although not having been explicitly discussed as an emotion regulation strategy so far, it aligns with the dominant definition of emotion regulation [21]: It influences how emotions are experienced and how they translate into behaviour.

Moreover, it shows certain parallels to established forms of emotion regulation. The first is mindfulness, with which it shares the non-judgemental awareness of emotions. However, critically differentiating IER and mindfulness, IER involves a much more active approach to emotions (see also [21]). A second family of emotion regulation strategies IER bears parallels with is what Gross [21] calls cognitive change. Both entail altering appraisals of (internal) situations, which alters the impact of the situation on the self.

A crucial difference between cognitive change strategies in the sense of reappraisal and the use of IER is the target of the cognitive operation. In the case of reappraisal, evaluations of a situation are the target. These are often altered in a way that allows one to “remain calm and objective” (e.g., [22,25]). In the case of IER, on the other hand, emotions are the target. These are cognitively transformed into valuable information (e.g., “My fear is neither good nor bad, it is legitimate and interesting”) that can be integrated with other aspects of the self. This newfound information can then be used to regulate one’s behaviour autonomously (which may include deliberately choosing further emotion regulation strategies; [24]). Hence, IER involves actively “working through” an emotional experience rather than distancing oneself from it, as would be the case when using emotional distancing (i.e., reappraising a situation in a way that allows remaining calm and objective; [25]). Further underscoring that reappraisal and IER might appear similar on a surface level, but have different correlates, research shows that interindividual differences in IER go beyond interindividual differences in reappraisal in predicting general sympathy and supportiveness towards outgroups [4].

Thus far, researchers have mainly focused on the intra- and interindividual benefits of IER. On an intraindividual level, IER is associated with "owning" negative experiences [26], less physiological stress responses to fear-eliciting stimuli, better cognitive functioning, and reduced self-reported fear at second exposure to the same stimulus [23,27]. On an interindividual level, IER correlates positively with sharing negative emotions with partners, supporting them in case of difficulties, more adaptive communication during conflicts with a partner [28,29], and greater supportiveness towards classmates [5].

First hints at the potential benefits of IER in the context of intergroup conflicts stem from two studies showing that interindividual differences in IER correlate with supportiveness towards outgroup members, mediated via sympathy [4]. Specifically, the more Israeli participants reported (chronically) using IER, the more sympathy and support for granting humanitarian aid to Palestinians they reported [4]. However, these studies do not shed light on how these relationships emerge. Closing this research gap, we propose and test two competing explanations: a person effect versus a person-situation interaction effect.

#### IER-sympathy: A person effect?

Individuals high in IER constantly actively attend to their emotions and where these come from. Moreover, they integrate the information they gain from doing so with their goals and values. Especially the latter may shape their general outlook on intergroup relations and specific outgroups. Specifically, integrating emotions felt towards an outgroup with goals and values appears highly conducive to the development of emotional sentiments. These are a "stable, general emotional disposition towards a person …[or] group" [30; p. 133] that result from repeatedly experiencing the same emotion towards the specific person or group. In case of IER, two processes could lead to these sentiments being characterised by sympathy.

First, individuals high in IER should be more likely to experience sympathy for others because they may extend the interested approach they take towards their own emotions to the emotions of others [5,24]. In terms of research on sympathy, this suggests that individuals high in IER should be more inclined and motivated to consider and understand other’s points of view, key predictors of feeling sympathy for them [31].

Second, their high awareness of their emotions should make individuals high in IER more likely to realize that they experience sympathy in the first place. After all, sympathy represents "other-oriented *feelings* congruent with the perceived welfare of another individual" [14, p. 621]). Taken together, this implies that individuals high in IER should be more likely to experience sympathy (because they take an interest in other’s emotions and situations) and more aware of feeling sympathy. Further contributing to solidifying the resulting prosocial emotional sentiments, IER may also inoculate individuals against adverse long-term effects of stimuli arousing negative emotions [24]. Given the previously demonstrated link between sympathy and supportiveness towards others (e.g., [13,19,20]; for a review, see [3]), we hypothesise the following:

*Person Hypothesis*: *The higher an individual is in IER*, *the more sympathy and supportiveness for a given outgroup they will express*.

Broadly in line with this idea, Roth and colleagues [4] found that interindividual differences in IER correlated positively with the general tendency to sympathise with others. At the same time, however, this measure of sympathy represents a limitation of Roth and colleagues’ work. On the one hand, it does not allow concluding whether IER is linked to higher sympathy for outgroups in particular. We address this issue by assessing sympathy with specific outgroups in the present work. On the other hand, it does not allow concluding how interindividual differences in IER might alter responses to specific situational circumstances that would typically make experiencing sympathy less likely.

#### IER-sympathy: A person-situation interaction?

For sympathy to arise, individuals need to be able and motivated to attempt to consider and understand another’s perspective. However, especially the motivation to "put oneself in outgroup members’ shoes" can be hampered by situational factors. These include enduring aspects like intergroup competition, high perceived group entitativity, asymmetries in power and access to valued resources, and zero-sum beliefs [32]. But also outgroup members’ behaviour in a situation can hamper sympathetic responses. Dovidio and colleagues [16], for instance, report on (unpublished) studies showing that participants are reluctant to sympathize with outgroup members who show reprehensible behaviour (i.e., attack a third person unprovoked). Johnson and colleagues [33] show that this sympathy-mitigating effect of negative behaviour by one outgroup member generalizes to the group as a whole: Individuals sympathized less with a member of an outgroup after another member of this group had behaved negatively towards participants’ ingroup. Three processes might underlie this effect: First, outgroup behaviour perceived as undesirable might make the outgroup as a whole appear less deserving of sympathy and help–a core appraisal underlying the experience of sympathy [34]. Second, such behaviour can induce negative emotions like anger or fear, which correlate negatively with sympathy [35,36]. Third, the behaviour in itself might be viewed as violating societal norms or threatening the ingroup, thereby escalating intergroup conflict [37]. In sum, one would expect individuals who face negative (i.e., undesired) outgroup behaviour to have a particularly low motivation to respond sympathetically to the offending outgroup–a situation likely characteristic of intergroup conflicts [17]. In line with appraisal frameworks of emotion and emotion regulation in intergroup conflicts [38], we propose that emotion regulation–in particular IER–might mitigate this sympathy-reducing effect of undesired outgroup behaviour.

Specifically, IER has been cast as a strategy that helps individuals to adaptively deal with their situational emotions [e.g., 6,7]. When facing outgroups, this should entail not falling prey to the negative action tendencies caused by outgroup members’ behaviour and the resulting negative emotions. Hence, those facing undesired outgroup behaviour should be less sympathetic and supportive towards outgroups (potentially because they experience negative emotions towards the outgroup), but only if they at the same time are low in IER. Being high in IER, in turn, should reduce this adverse effect of negative outgroup behaviour (potentially due to a down-regulation of negative emotions). This train of thought implies that the IER-sympathy link emerges as a person-situation interaction.

*Person-Situation Interaction hypothesis*: *IER will mitigate the adverse effects of an outgroup’s undesired behaviour (vs a control condition) on sympathy and supportiveness towards members of this outgroup*.

Looking at the expected result pattern another way suggests that when outgroup behaviour is neutral, thus posing no barriers to responding with sympathy, reported sympathy and supportiveness towards an outgroup should be independent of interindividual differences in IER. Only when situational circumstances represent barriers to sympathetic responding should these differences matter, with those high in IER responding more sympathetically than those low in IER.

### The current research

Given that sympathy is a key correlate of intergroup forgiveness and reconciliation and plays an important role in intergroup conflicts [17,32,36,39], understanding who is most likely to sympathise with outgroup members (and when) contributes to our understanding of the benefits emotion regulation might have for promoting positive intergroup relations beyond the mere downregulation of negative emotions. Herein, we take a step towards answering the question of whether reaping the benefits of IER in terms of higher sympathy is either a question of “who” (i.e., a Person effect) or of “who and when” (i.e., a Person x Situation effect).

As Roth and colleagues [4] assessed supportiveness for an outgroup without referencing outgroup behaviour, their results can only speak to the validity of the person hypothesis. This is problematic because effects regarding interindividual differences in emotion regulation do not necessarily inform about emotion regulation in a specific situation [40,41]. Consequently, the existing evidence leaves open whether interindividual differences in IER shape reactions to out-groups whenever (negative) emotions arise or colour people’s general view of an outgroup. In other words, it is unclear whether the correlations between IER, sympathy and supportiveness result from (a) the regulation of emotions elicited in the situation at hand or (b) more generalised patterns that could, for instance, result from the integration of experiences to form long-term impressions. In the present work, we tested which of these two possible explanations—a Person x Situation interaction vs a Person effect–is the more plausible explanation for the link between IER and supportiveness. This required us to assess the correlations of IER with sympathy and outgroup-directed supportiveness (a) with regard to a specific outgroup and (b) in specific emotion-eliciting situations. We did so in four main experiments involving different outgroups (e.g., refugees, older people, drug addicts, homeless people). This allowed us to test whether the positive IER—supportiveness relation found by Roth and colleagues [4] generalises across intergroup contexts. In an additional study, we did not successfully manipulate emotions. Therefore, this study did not allow for adequately testing the Person-Situation Interaction Hypothesis and is reported in the Supplement only (see S1 File).

More importantly, it allowed us to test the two competing explanations for this association outlined above. This required two deviations from previous research on the relations between IER, sympathy, and supportiveness in intergroup contexts. First, we confronted different participants with different behaviours by members of the same outgroup. According to Čehajić-Clancy and colleagues [15], anger is likely to emerge when outgroup behaviour is viewed as unjust, unfair, and deviating from acceptable norms. Fear, in turn, is likely to emerge when the outgroup’s behaviour threatens the ingroup. Consequently, some participants in our studies received information about or recalled undesired (i.e., non-normative or threatening) behaviour by outgroup members (e.g., fraud committed, violence displayed, littering). Others received factual information about the group, information about behaviour that was not undesired or recalled such behaviour. Second, we asked participants to report their sympathy for the outgroup targeted in our behaviour manipulation. This “targeted” sympathy is more proximally related to supportiveness towards the specific outgroup. Therefore, it should be a better predictor than dispositional sympathy assessed in earlier studies. Of note, independent of whether the IER-sympathy link would be better explained by the Person or the Person-Situation Interaction Hypothesis, we expected sympathy to mediate the effect of IER (or the IER x Behaviour interaction) on supportiveness towards the outgroup.

#### Ethics and open science

The present work received ethical approval from the IRB at the Leibniz-Institut für Wissensmedien, Tübingen, and written informed consent was obtained from all participants, either by participants signing a form (in case of lab studies) or by participants clicking a button indicating their consent after they had received all pertinent study information. We report all manipulations and exclusions as well as how sample size was determined. Data, code, and study materials are available at: https://doi.org/10.23668/psycharchives.14037 (Study Materials), https://doi.org/10.23668/psycharchives.14038 (data), and https://doi.org/10.23668/psycharchives.14036 (code). We used IBM SPSS Statistics (Version 25, [42]) for our analyses if not indicated otherwise and set the significance threshold to 5% for all analyses.

We preregistered Studies 1 (https://aspredicted.org/cj9i8.pdf), 3 (https://aspredicted.org/7af82.pdf), and 4 (https://aspredicted.org/p8rb8.pdf) as tests of the two hypotheses reported herein. Study 2 was preregistered to test a different hypothesis (see S1 File). Pre-registrations for Studies 1, 3, and 4 include study design, analyses, and planned sample size. We varied pre-registered inclusion criteria slightly between studies due to differences in methods and did not adhere to them in the following cases: In Study 1, we excluded additional participants due to a change in procedures (i.e., participants who completed our IER-measure after the behaviour manipulation, were refugees, or skipped the manipulation); in Study 3, we excluded neither participants who took twice the mean time to complete the survey nor outliers; in Study 4, we excluded non-compliant participants. Across studies, analyses with the full samples revealed result patterns identical to those reported below. Interested readers can find the results of these analyses in the (S1 File). This file also contains the results of any preregistered analyses not reported herein.

For each study, we report the basis of our preregistered sample size planning below. Hence, analysis procedures described as the basis for our a priori power analyses do not always correspond to the analyses used in the respective studies. Therefore, all a priori power analyses are supplemented with a sensitivity power analysis based on the final sample in each study.

## Studies 1A/B

We conducted this study after the so-called refugee crisis in Germany in 2015/16. At the time of data collection, political debates on the number of accepted refugees were still ongoing. Especially parties on the far-right branded refugees as potential criminals and terrorists, meaning that the present study’s context was very politicised. To create the necessary conditions for testing our Person-Situation Interaction Hypothesis, in this study we confronted participants with outgroup behaviour assumed to elicit anger, fear, or no specific emotions towards the outgroup.

### Method

#### Participants & design

This study had a fully between 3 (outgroup behaviour: undesired-fraud, undesired-terrorist attacks, control) x continuous (measured IER) design. We based our a priori power analysis for sample size planning on the zero-order correlations between IER and sympathy reported by Roth and colleagues [4]. This suggested powering the study to detect a small-to-medium (overall) effect (*f*^2^ = 0.10) with 90% likelihood (α = 5%). Running this analysis with G*Power [43] for a linear multiple regression with seven predictors (as we erroneously planned to include interactions between the two orthogonal contrasts as well), suggested collecting 190 responses. As we failed to reach this sample size within one data collection timeslot in our lab, we re-started data collection in a separate slot and combined the two resulting data sets (not preregistered). Data collection for slot A took place from 05/02/2018-05/18/2018 and for slot B from 06/05/2018-06/15/2018.

Of the 369 German university students who participated in the two data collection slots, we lost 22 (two due to data withdrawal, 20 due to issues matching their online data to their lab data). Moreover, we excluded 91 participants distributed equally across conditions: Eight completed the IER measure after the debriefing or were refugees, one skipped the manipulation (both not preregistered, but necessary to ensure comparability), 55 in the second slot reported prior participation in a similar experiment (which could have been the same study in the first slot, leading to familiarity with the materials), 23 reported that German was not among their native languages (as we were interested in the relation between Germans and refugees), and four were outliers (SDR > ± 2.65 in a regression with supportiveness as the dependent variable and IER, the two contrasts, and the relevant interactions as predictors). Thus, we analysed data from 256 participants (M_age_ = 22.84, 18–34, 50 male, 203 female, 3 participants did not indicate gender; for analyses with the full analysable sample, see Statistical Supplement). A sensitivity analysis with G*Power [43] suggests that this sample is large enough to detect, with 80% likelihood, an increase in R^2^ by f^2^ = 0.03 (r = 0.17) caused by one predictor in a linear multiple regression with a total of five predictors, reflecting the analysis conducted in this study.

#### Procedure

We programmed and delivered the study via empirisoft medialab [44]. We obtained written informed consent before the study began. In data collection slot 1A, participants completed the IER measure online before coming to the lab. The lab session consisted of an independent first study and the study described herein. In data collection slot 1B, participants completed the IER measure at the beginning of the lab session. Afterwards, two independent studies were run before our study started. In both data collection slots, participants who underwent different manipulations in the prior studies were distributed equally across the conditions of the present study. The focal result pattern was not contingent on the data collection slot.

According to a pre-defined list, participants were randomly assigned to watch one of three ca. 4.5min long film clips. Each clip consisted of snippets from multiple clips from various news outlets that were cut and combined for the present research’s purposes (for details, see Study Materials at https://doi.org/10.23668/psycharchives.14037). In the *control condition*, the clip discussed prejudice against refugees and information disconfirming those prejudices. In the *"undesired behaviour—fraud"*-condition, the clip reported on identity fraud committed by refugees to obtain several social welfare payments per month and violent outbreaks in a housing facility for refugees attributed to a lack of wireless internet. It was intended to elicit anger (see S1 File). The clip in the *"undesired behaviour—terror"*-condition consisted of several short clips on ISIS smuggling terrorists into Germany disguised as refugees as well as terrorist attacks in Germany and Paris. It was intended to elicit anxiety (for details, see S1 File).

After the film clip, we assessed participants’ emotions, our focal outcome variables and several exploratory measures (for details, see Study Materials). Next, we collected demographic information, measures of participants’ political attitudes, and information on familiarity with the film clips. Finally, participants were debriefed and received 10€ (slot A) or 8€ (slot B).

#### Measures

If not indicated otherwise, we assessed all variables on seven-point scales ranging from 1 ("don’t agree") to 7 ("agree").

*IER*. IER was assessed with six items (a = .81) based on a German translation of the scale developed by Roth and colleagues [6]. The items were "When I feel tense or anxious, I try to find out what this tells me about myself and the situation I am in.", "Sometimes my tension and fear have helped me to better understand the situations I was in.", "There have been situations in which it helped me to talk about my fear and my tension.", "When I feel anxious or tense, it is important to me to find out why I feel this way.", "I often think about my fears and apprehensions to understand what causes them." and "When I feel anxious and tense, I normally try to understand the reasons why I feel this way.".

*Elicited Emotions*. Participants reported how they felt after watching the film clips (1 = "not at all"; 7 = "very") based on four items: angry, anxious (both as checks for our behaviour manipulation), interested, and calm (both included as distractors).

*Sympathy*. Six items (a = .91) measured participants’ sympathy towards refugees, e.g., "When I think about refugees, I am often compassionate". Four of these items were adapted from Batson [45]; two items were adapted from de Vos and colleagues [46].

*Supportiveness*. We used nine self-developed items (a = .79: e.g., "Refugees should be granted full access to the German health care system.") to assess how strongly participants favoured various policies benefitting refugees.

### Results

For analyses, we z-standardized IER and contrast coded our experimental factor (Desirability-contrast: control condition -2, undesired behaviour conditions 1; Residual contrast: control condition 0, terror -1, fraud 1).

#### Elicited emotions

A one-factorial ANOVA showed that, as expected, the clips reporting undesired behaviour elicited more negative emotions than the control clip (for descriptives, correlations, and statistics on a condition main effect, see Table 1). Specifically, the terror clip elicited more *anxiety* than the fraud (*p* = .001) and the control clip (*p* < .001) and the fraud clip elicited more *anger* than the terror clip (*p* = .011). Moreover, the fraud clip elicited more anxiety (*p* < .001) and the terror clip more anger (*p* < .001) than the control clip. Thus, our behaviour manipulation appears to have been successful.

**Table 1:** Descriptive statistics, ANOVA results for condition main effect, and correlations between measures, *N* = 256, Study 1.

	control *M(SD)* *n* = 85	fraud *M(SD)* *n* = 88	terror *M(SD)* *n* = 83	*F*(2,253)	*p*	h_p_^2^ *CI*_*90%*_ (LL, UL)	2.	3.	4.	5.
1. Anxious	1.48 (1.03)	2.59 (1.45)	3.35 (1.69)	37.14	< .001	.227 (.152, .294)	.37***	-.001	-.11^†^	-.29***
2. Angry	3.25 (1.85)	5.28 (1.42)	4.64 (1.66)	34.15	< .001	.213 (.139, .279)		.12^†^	.07	-.13*
3. IER	5.00 (1.17)	5.15 (1.04)	5.04 (1.11)	0.44	.648	.003 (0, .019)			.14*	.07
4. Sympathy	5.15 (1.11)	5.29 (1.14)	5.07 (1.12)	0.85	.428	.007 (0, .027)				.62***
5. Support.	5.20 (0.90)	4.89 (1.01)	4.93 (0.97)	2.62	.074	.020 (0, .053)				-

support. = supportiveness, LL = lower limit of 90% confidence interval, UL = upper limit of 90% confidence interval

*** *p* < .001

* *p* < .05

^†^ *p* < .10.

#### Hypotheses tests

As shown in Table 2, this study yielded partial support for our *Person-Hypothesis*. For sympathy, we found the main effect that we would expect if IER coloured respondents’ general outlook on the outgroup. This was not the case for supportiveness. Supportiveness was only affected by our behaviour manipulation: Participants were less supportive of refugees in the undesired behaviour conditions than in the control condition (desirability contrast).

**Table 2:** Results of regression analyses, Study 1; *N* = 256. Main effects were tested first, with interactions added in a second step.

Criterion	Predictor	*B*	*SE*	β	*t* ^*^	*p*	*CI*_*95%*_ *(B)*	
							*LL*	*UL*
Sympathy	IER	0.16	0.07	0.14	2.21	.028	0.02	0.29
	Desirability Con.	0.004	0.05	0.01	0.09	.929	-0.09	0.10
	Residual Contrast	0.10	0.09	0.08	1.20	.230	-0.07	0.27
	IER x Des. Con.	-0.05	0.05	-0.07	-1.05	.295	-0.15	0.04
	IER x Res. Con	-0.04	0.09	-0.03	-0.46	.647	-0.21	0.13
Supportiveness	IER	0.08	0.06	0.08	1.29	.198	-0.04	0.20
	Desirability Con.	-0.10	0.04	-0.14	-2.31	.021	-0.18	-0.02
	Residual Contrast	-0.03	0.07	-0.02	-0.38	.703	-0.17	0.12
	IER x Des. Con.	0.04	0.04	0.07	1.05	.294	-0.04	0.13
	IER x Res. Con	-0.01	0.08	-0.01	-0.17	.865	-0.16	0.14

LL = Lower Limit; UL = Upper Limit; Des. Con. = Desirability contrast; Res. Con. = Residual contrast.

*df for main effects: 252; df for interactions: 250.

However, the IER x Behaviour interaction effect expected based on the *Person-Situation Interaction Hypothesis* was non-significant for both dependent variables. Thus, the present findings do not support the idea that IER is particularly beneficial when extraneous circumstances (e.g., an outgroup’s undesired behaviour) would normally reduce the likelihood of individuals responding prosocially. In line with the interactions proving non-significant, the main effects remained unchanged when including the interactions as predictors.

Finally, we tested whether sympathy mediates the relationship between IER and supportiveness. Deviating from our preregistration, we used PROCESS model 4 (v 3.5;[47]) instead of 8, as our previous analyses did not suggest a moderation. Replicating previous research [4], the indirect effect of IER on supportiveness via sympathy was significant, *B* = 0.08, *SE*_*Boot*_ = 0.04, *CI*_*95%*_[0.01, 0.16]. This suggests that IER is indirectly related to supportiveness towards outgroup members through greater sympathy.

### Discussion

The current findings suggest that IER positively relates to sympathy and (descriptively and indirectly) supportiveness towards outgroups, supporting our Person-Hypothesis. In contrast to our Person-Situation Interaction Hypothesis, IER did not moderate the effect of undesired (vs neutral) outgroup behaviour on our dependent variables. Given the merely indirect effect of IER on supportiveness, however, this evidence is preliminary. Therefore, we tested our hypotheses again in Study 2, which also addresses three issues of Study 1.

First, one might suspect that we did not find the predicted interaction on sympathy and supportiveness because our participants were already highly sympathetic and supportive towards refugees (as indicated by the high overall means in both variables; sympathy: *M* = 5.17, *SD* = 1.12; supportiveness: *M* = 5.00, *SD* = 0.97), which might also have been part of their political identity. Therefore, we focused on a different group and a less politically charged intergroup conflict in Study 2. Second, the content of the films might have been too abstract to cause emotions that reduce participants’ general positive tendencies toward refugees. Study 2, therefore, used vignettes describing more relatable experiences, as this may cause more personally relevant emotions. Finally, as we had to exclude many participants in this study, we conducted Study 2 online and in one go. Moreover, given that we found no differences in sympathy and supportiveness between the two undesired behaviour conditions, we do not further address differences in the effects of anger- or anxiety-inducing behaviour. Please note that Study 2 was originally intended to test a different hypothesis, but its design and results allow for testing and correspond to the focal hypotheses presented in this manuscript.

## Study 2

### Method

#### Participants & design

The IER-sympathy relationship found in Study 1 was smaller than in prior research. Therefore, we dropped our estimate of the effect size to *f*^2^ = 0.085 for our a priori power analysis in G*Power ([43]; linear multiple regression, four predictors, α = 5%, 1-β = 90%), which yielded an ideal sample size of 187.

Two hundred and five participants completed all necessary variables in this fully between 2 (undesired behaviour vs control) x continuous (measured IER) online experiment during the data collection period (06/27/2019-06/28/2019). One participant withdrew their data after the debriefing. We excluded 26 participants according to preregistered criteria, distributed roughly equally across conditions: Five were suspicious about the study aim, two were unrealistically fast (i.e., took less than 3min to complete the survey), four were older than 35 (materials were designed for typical students), and thirteen participants studied psychology (excluded due to potential familiarity with the scales used in the present study). Based on our cut-off criterion for outliers from Study 1, we excluded two outliers. Thus, analyses are based on 178 participants (*M*_age =_ 22.97, 18–35; 45 male, 131 female, two diverse). A sensitivity analysis with G*Power [43] suggests that this sample is large enough to detect, with 80% likelihood, an increase in R^2^ by f^2^ = 0.04 (r = 0.21) caused by one predictor in a linear multiple regression with a total of three predictors, reflecting the analysis conducted in this study.

#### Procedure

The online questionnaire was generated and delivered using SoSci Survey [48]. After obtaining informed consent, we assessed IER. Then, we randomly assigned participants to one experimental condition (aiming for equal distribution in finished questionnaires) and instructed them to imagine commuting to work.

Both conditions described encounters with several older persons. In the *undesired behaviour* condition, they drove slowly, got into participants’ lane and reacted belatedly, or tottered slowly across the street. In the *control* condition, they drove the speed limit, reacted quickly when getting into participants’ lane, and hurried across the street.

Afterwards, we assessed sympathy and supportiveness for older people. Next, we gave a short extension of the vignette, which described a misfortune befalling an older lady, assessed participants’ willingness to help her and collected two further variables, demographics (including how frequently respondents drive) and suspicions about the study content. Finally, participants provided information on previous participation in similar studies, could leave comments, were debriefed and could participate in a raffle as compensation.

#### Measures

If not indicated otherwise, all measures were collected on 7-point Likert scales from 1 ("don’t agree at all") to 7 ("fully agree").

*IER*. We added two items to the scale used in Study 1 based on the third author’s revision of the original scale (see Study Materials). The scale then consisted of eight items (a = .86).

*Sympathy*. We assessed sympathy with the same items used in Study 1, but changed the target to "older people", a = .87.

*Supportiveness*. We assessed supportiveness with six items tapping into participants’ support for policies benefitting older persons and their willingness to act on behalf of older persons. We dropped one item ("I cannot imagine living in a multi-generation house and helping older housemates with chores.", inverse scored) from the scale due to a low item-scale correlation, *r* (178) = .10. The remaining five items formed the supportiveness scale, a = .73.

### Results

To test our hypotheses, we regressed our dependent variables on IER (z-stand.), behaviour (-1 = control, 1 = undesired behaviour), and the IER x Behaviour interaction (for descriptives by condition and condition effects, see Table 3).

**Table 3:** Descriptive statistics, independent samples t-test results (two-tailed), and correlations between measures, *N* = 178, Study 2.

	Control *M(SD)* *n* = 92	UDB *M(SD)* *n* = 86	*t*(176)	*p*	*M*_*Diff*_	*CI*_*95%*_ *[M*_*diff*_*]*		2.	3.
						LL	UL		
IER	4.82(1.14)	4.73(1.12)	0.54	.593	0.09	-0.24	0.43	.32***	.37***
sympathy	4.78(1.19)	4.33(1.09)	2.62	.010	0.45	0.11	0.79		.52***
supportiveness	4.58(1.15)	4.40(1.10)	1.03	.306	0.17	-0.16	0.51		-

UDB = Undesired Behaviour, LL = Lower Limit, UL = Upper Limit^.^

*** *p* < .001.

In line with the *Person-Hypothesis*, we found significant IER main effects on sympathy and supportiveness (see Table 4), indicating that those higher in IER reported more sympathy and supportiveness towards older persons. In addition, behaviour significantly affected sympathy, but not supportiveness: Participants reported less sympathy for older persons when they had imagined them showing undesired behaviour. Contrary to our *Person-Situation Interaction Hypothesis*, however, the IER x Behaviour interaction affected neither sympathy nor supportiveness. Results of our exploratory analysis were broadly in line with this pattern, showing a positive link between IER and participants’ situation-specific supportiveness towards an older lady (for details, see S1 File).

**Table 4:** Results of regression analyses, Study 2, *N* = 178.

Criterion	Predictor	*B*	*SE*	β	*t* ^*^	*p*	*CI*_*95%*_ *(B)*	
							*LL*	*UL*
Sympathy	IER	0.37	0.08	0.32	4.48	< .001	0.21	0.53
	Behaviour	-0.21	0.08	-0.18	-2.57	.011	-0.37	-0.05
	IER x Behaviour	-0.06	0.08	-0.05	-0.75	.456	-0.22	0.10
Supportiveness	IER	0.41	0.08	0.37	5.24	< .001	0.26	0.57
	Behaviour	-0.07	0.08	-0.06	-0.89	.375	-0.23	0.09
	IER x Behaviour	-0.04	0.08	-0.03	-0.48	.629	-0.19	0.12

LL = Lower Limit, UL = Upper Limit.

*df for main effects: 175; df for interaction: 174.

Finally, we again ran a mediation analysis analogous to the previous study. In line with our prediction and prior work [4], we found that IER relates indirectly to supportiveness via sympathy, *B* = 0.16, *SE*_*Boot*_ = 0.04, *CI*_*95%*_[0.09, 0.26].

### Discussion

Partially replicating Study 1, the present results again did not support the Person-Situation Interaction Hypothesis but rather the Person-Hypothesis: IER again did not moderate the effects of the outgroup’s behaviour on sympathy or supportiveness but correlated positively with both variables. Mediation analyses further showed that IER was positively related to supportiveness through higher levels of sympathy. Our findings also fit the idea that undesired behaviour may hamper sympathetic responding and that emotions guide intergroup behaviour. Specifically, participants in the undesired behaviour condition (that presumably caused anger) were less sympathetic towards older persons.

In sum, the present study again suggests that the relations between IER, sympathy, and supportiveness generalise across different groups. We sought to replicate this finding in Study 3 and further investigate its generalizability by confronting each participant with members of six different groups showing (vs not showing) undesired behaviour.

## Study 3

### Method

#### Participants and design

To determine sample size, we ran two a priori power analyses using G*Power ([43]; analysis type "ANOVA: Repeated measures, within-between interaction"; input: α = %5, 1-β = 90%, number of groups = 2, number of measurements = 6, corr. among rep. measures = 0.5, nonsphericity correction = 1), one for a medium effect (*f* = 0.25, suggesting *N* = 40) and one for a small effect (*f* = 0.10, suggesting *N* = 140), as the differences in design prevented us from using the effect sizes obtained in the previous studies as a basis for effect size estimation. Based on the results, we aimed to collect 200 responses to retain a sufficiently large sample even after necessary exclusions. Data collection took place between 07/22/2019 and 07/23/2019.

In total, 206 participants completed this online survey, which had a 2 (outgroup behaviour: control vs undesired; within participants) x continuous (measured IER; between participants) mixed design. To manipulate outgroup behaviour, we showed each participant six vignettes involving different outgroups. In three of these, the described outgroup member showed undesired behaviour; in the other three, they did not. We formed two sets of vignettes (Set A: undesired behaviour by groups A, B, and C; Set B: undesired behaviour by groups D, E, and F; see below) to control for differences between the outgroups as a potential confound and randomly assigned one set to each participant as in Study 2 (for further information on the effect of material set, see Supplement).

One participant withdrew their data after the debriefing. Based on our preregistered exclusion criteria, we excluded 29 participants: Eight took less than half the mean time to complete the study, four voiced suspicions regarding the study aim, ten indicated that German was not among their native languages (problematic because two vignettes involved migrants as an outgroup), and seven were older than 35 (problematic because materials were designed for students). Deviating from our preregistration, we did not exclude participants who took twice the mean time to complete the survey, as each vignette can be considered a manipulation of its own. Moreover, we did not exclude outliers to retain equal cluster sizes. Thus, we analysed data from 176 participants (*M*_age =_ 23.54, 18–35; 46 male, 125 female, three diverse; two did not provide information on gender, and one did not provide valid information on age). We further deviated from our preregistration by not controlling for effects of the specific outgroup on our dependent variables, as this effect would have been confounded with outgroup behaviour. A sensitivity analysis via Monte Carlo simulations using Mplus [49] suggests that this sample is large enough to detect, with 80% likelihood, a cross-level main effect of a between person variable on the between-person variance in a Level 1 variable of *B* = 0.22 (*r* = 0.22), reflecting the analysis conducted in this study.

#### Procedure

Participants were invited via email to participate in this online study which again was programmed and delivered via SoSci Survey [48]. Participants first provided informed consent and information on their emotion regulation. Then, they read the vignettes, in which they were observers, in random order.

The outgroups used in the vignettes were (implicitly) refugees (Group A), homeless people (Group B), social welfare recipients (Group C), drug addicts (Group D), older people (Group E), and Turks (Group F; for behaviours by condition, see Table 5).

**Table 5:** Outgroup behaviour by group and condition.

Outgroup	Undesired Behaviour	Behaviour in control condition
refugees	group of Arabic-speaking men stealing someone’s purse	group of Arabic-speaking men asking someone for a lighter
Homeless people	homeless man harassing visitors in a park	homeless man calmly asking for change
Social welfare recipients	woman complaining about cuts to her welfare payments because she did not accept a job not paying more than her social welfare payments	woman mumbling about being unsure which documents she had forgotten to submit
Drug addicts	people in a spot frequented by drug addicts throwing used syringes into a hedge where children play during the day	people in a spot frequented by drug addicts talking to each other
Older people	older couple ramming another shopper at the checkout with their cart and bickering	older couple carefully arranging their shopping on the conveyor belt
Turks	group of people littering plastic cups and plates in the forest during a barbecue	group of people picking up paper napkins that had been blown away during their barbecue

After each vignette, we assessed sympathy and supportiveness towards the respective group and elicited emotions. Finally, we collected several additional measures (see S1 File and Study Materials) and demographic data, including political orientation, and asked participants about their suspicions, remarks, and participation in similar previous studies. Finally, participants were thanked, debriefed, could withdraw their data, and could participate in a raffle for vouchers.

#### Measures

*IER*, *Sympathy and Elicited Emotions*. We used scales similar to Study 2 to assess IER (focusing on anxiety; a = .88) and sympathy (only adding "often" to the items as in Study 1). The target of sympathy was adapted to the content of the vignette (a = .84 - .93). Elicited Emotions were assessed analogous to Study 1.

*Supportiveness*. We used five items per group (a = .72 - .81) to assess supportiveness, operationalized as participants’ agreement with statements about their willingness to donate and volunteer for an outgroup and about the need for more societal support for the outgroup. Scale anchors were 1 ("do not agree) and 7 ("agree").

### Results

We analysed this study using a multilevel approach, as responses to vignettes were nested within participants (i.e., participants represented the clustering variable). We entered IER (only varying between participants, i.e., clusters) as a manifest Level 2 variable and behaviour (contrast coded as -1 = control and 1 = undesired behaviour and varying within participants) as a manifest Level 1 variable. Following Preacher and colleagues’ [50] suggestions, we decomposed the variance in sympathy and supportiveness into a within-part and a between-part by modelling them on Levels 1 and 2. Thereby, we could account for the fact that sympathy and supportiveness should vary within participants (i.e., between vignettes) but may *also* vary between participants (e.g., due to some people generally being more sympathetic and supportive than others).

We modelled the effect of behaviour on the outcomes at the within-level and the IER x Behaviour interaction (predicted by our Person-Situation Interaction Hypothesis) as a cross-level interaction. To this end, we first modelled the effect of behaviour on the within-person variance of sympathy and supportiveness as random slopes. Then, we entered IER as a Level 2 predictor of these random slopes following a random coefficient prediction logic. We chose this approach as the Level 1 predictor—behaviour—had no Level 2, between-person variance. We used MPlus (version 8.1, 41) for all analyses (analysis-type "twolevel random", estimator: MLR).

#### Elicited emotions

We tested whether participants reported more negative emotions when faced with undesired (vs not undesired) behaviour in a multilevel analysis. Behaviour significantly affected anxiety and anger. Participants reported more negative emotions in response to undesired than not undesired behaviour, confirming the success of our manipulation (see Table 6).

**Table 6:** Results of multilevel moderation analysis, *N* = 176.

Criterion	Predictor	*B*	*SE*	*z*	*p*	*CI*_*95%*_ *[B]*	
						LL	UL
Anger	Behaviour	1.55	0.05	31.44	< .001	1.45	1.64
Anxiety	Behaviour	0.40	0.03	11.47	< .001	0.33	0.46
Sympathy	Behaviour	-0.06	0.04	-1.46	.146	-0.15	0.02
	IER	0.32	0.08	3.99	< .001	0.16	0.47
	IER x Behaviour	-0.02	0.05	-0.48	.631	-0.12	0.07
Supportiveness	Behaviour	-0.06	0.03	-2.31	.021	-0.11	-0.01
	IER	0.22	0.07	3.12	.002	0.08	0.36
	IER x Behaviour	0.001	0.03	0.05	.963	-0.05	0.05

LL = Lower Limit, UL = Upper Limit.

#### Hypotheses tests

Our Person-Situation Interaction hypothesis predicted a cross-level interaction, namely that IER (varying only between participants) would moderate the effect of behaviour (varying within participants) on sympathy and supportiveness. This hypothesis again received no support (see Table 6). Instead, we found the positive IER main effect on (between-person differences in) both dependent variables predicted by our Person-Hypothesis. That is, those who scored higher in IER also reported more sympathy and supportiveness, irrespective of portrayed outgroup behaviour. Outgroup behaviour did, however, affect supportiveness in that participants were less supportive towards outgroups when they had (vs had not) read descriptions of undesired behaviour.

As a final step, we tested whether sympathy mediated the effect of IER on supportiveness, following the multilevel structural equation modelling (MSEM) approach suggested by Preacher, Zhang and Zyphur [50]. We regressed *intra*individual differences in supportiveness on *intra*individual differences in sympathy, *inter*individual differences in supportiveness on *inter*individual differences in sympathy, and *inter*individual differences in both variables on IER. Note that IER could only be entered as a predictor of the between-person relations because it did not vary within participants (for a conceptual model, see Supplement).

Results reveal that within participants, sympathy positively relates to supportiveness, *B* = 0.55, *SE* = 0.03, *z =* 19.53, *p* < .001, *CI*_*95%*_[0.50, 0.61]. Between participants, sympathy is related to IER, *B* = 0.32, *SE* = 0.08, *z =* 3.99, *p* < .001, *CI*_*95%*_[0.16, 0.47], and in turn predicts supportiveness, *B* = 0.74, *SE* = 0.09, *z =* 8.36, *p* < .001, *CI*_*95%*_[0.57, 0.92]. IER does not have a direct effect on supportiveness, *B* = -0.01, *SE* = 0.06, *z =* -0.20, *p =* .845, *CI*_*95%*_[-0.13, 0.11]. Importantly, the indirect effect of IER on supportiveness via sympathy on the between-person level is significant, *B* = 0.23, *SE* = 0.06, *z =* 3.83, *p* < .001, *CI*_*95%*_[0.11, 0.35]. This pattern is in line with the two previous studies and implies that individuals high in IER express more supportiveness, mediated via sympathy (i.e., IER "explains" *inter*personal differences in supportiveness mediated via *inter*personal differences in sympathy).

### Discussion

Even using a within-participants design and multiple outgroups, we only found support for the Person Hypothesis but not the Person-Situation Interaction Hypothesis: Interindividual differences in IER were positively related to (*inter*individual differences in) sympathy and supportiveness. However, IER did not interact with the outgroup’s portrayed behaviour in predicting differences in sympathy or supportiveness between vignettes (i.e., *intra*individual differences in sympathy or supportiveness). One might speculate that this is due to our manipulations not eliciting self-relevant emotions that require regulation, meaning that there is no chance for the Person-Situation interaction to emerge. Even though the effects of our manipulation on emotions reported by participants speak against this explanation, we decided to ensure self-relevance of the outgroup’s behaviour in Study 4 by using a guided recall approach.

## Study 4

The key goal of this study was to ascertain whether the lack of support for the Person-Situation Hypothesis in prior studies was due to a lack in self-relevance of the elicited emotions. A secondary goal of this study was to test whether IER explains variance in sympathy and supportiveness beyond other forms of emotion regulation. While IER shares conceptual similarities with both reappraisal and mindfulness, evidence that it goes beyond the former already exists [4], whereas evidence of it going beyond mindfulness is, to date, lacking. Thus, we focused on mindfulness in our study, simultaneously testing whether IER also goes beyond the Big 5. Details regarding this analysis are available in the S1 File.

### Method

#### Participants & design

This preregistered study had a 2 fully between (recalled behaviour: undesired vs control) x continuous (measured IER) design. We collected the data from 03/28/2022 to 04/04/2022. To manipulate behaviour, participants recalled an interaction with an older person that had made them angry (undesired behaviour) or was normal for them (no undesired behaviour).

Based on our secondary goal, we followed recommendations by Westfall and Yarkoni [51] and aimed to collect 650 responses. After one week, we had managed to collect 439 responses. Given a strong decline in response rate, we opted to close the survey at this point. We programmed and delivered the experiment via www.qualtrics.com, recruited participants from Prolific and paid them approximately £7.95/h.

Following our preregistered criteria, we excluded 65 participants (control condition *n* = 39; undesired behaviour *n* = 26): 22 did not pass the two attention checks, four reported not having completed the survey in one go, two reported having completed the study multiple times, 23 participants in the control condition recalled situations involving undesired behaviour or described negative emotions in the situation, and nine participants did not describe a concrete situation (not preregistered). Furthermore, we excluded five outliers according to the criterion used in Studies 1 and 2. None of the remaining participants fulfilled our other exclusion criteria. Thus, all analyses are based on 374 observations (*M*_age =_ 25.59, 18–35; 159 male, 208 female, six diverse, one other). As preregistered, the IER-sympathy and the IER-supportiveness relationships held even when controlling for trait mindfulness and the Big5 (excluding agreeableness due to its compassion-facet). Given that the sample size is not in line with what is recommended for such analyses (but clearly sufficient for our main goal), we report these results in the Statistical Supplement (see S1 File). A sensitivity analysis with G*Power [43] for our focal analysis suggests that our sample is large enough to detect, with 80% likelihood, an increase in R^2^ by f^2^ = 0.02 (r = 0.14) caused by one predictor in a linear multiple regression with three predictors, reflecting the analysis conducted in this study.

#### Procedure

We first obtained informed consent and measured mindfulness, personality traits (for details, see Supplement), and IER. Next, participants were randomly and evenly assigned to one of the two experimental conditions. In the *control* condition, we asked them to recall an interaction with an older person that is commonplace for them. In the *undesired behaviour condition*, we participants to recall an interaction with an older person that had made them angry. In both conditions, participants received some examples of situations to make the task easier for them (for details, see Study Materials). Then, we assessed how participants had felt during the recalled situation and our dependent variables. Finally, participants provided demographic information and could leave comments on the study before being debriefed and thanked.

#### Measures

*IER*, *Sympathy*, *Supportiveness*. We used the same items as in Study 3 to assess IER (a = .88), sympathy (a = .92), and supportiveness towards older people (a = .67).

*Experienced Emotions*. We added two items ("outraged" and "content") to the four used in Studies 1 and 3 to assess how participants had felt during the recalled interaction. We averaged participants’ responses to the items "outraged" and "angry", *r*(374) = 0.93, to form our anger manipulation check.

### Results

#### Experienced emotions

We expected that participants would report more anger in the undesired vs the no undesired behaviour condition. An independent samples t-test (two-tailed) confirmed this expectation (see Table 7). Thus, our manipulation of the behaviour’s desirability was successful.

**Table 7:** Descriptive statistics, independent samples t-test results (two-tailed), and correlations between measures, *N* = 374, Study 4.

	Control *M(SD)* *n* = 179	UDB *M(SD)* *n* = 195	*t*(372)	*p*	*M*_*Diff*_	*CI*_*95%*_ *[M*_*diff*_*]*		2.	3.	4.
						LL	UL			
Anger	1.25(0.74)	5.48(1.43)	-36.27^a^	< .001	-4.23	-4.46	-4.00	.03	-.12*	-.20***
IER	4.52(1.18)	4.61(1.08)	-0.74	.463	-0.09	-0.32	0.14		.20***	.21***
sympathy	4.87(1.22)	4.56(1.23)	2.42	.016	0.31	0.06	0.56			.54***
supportiveness	4.88(0.91)	4.52(0.97)	3.70	< .001	0.36	0.17	0.55			

UDB = Undesired Behaviour, LL = Lower Limit, UL = Upper Limit.

*** *p* < .001

* *p* < .05

^†^ *p* < .10.

^a^df anger = 296.53.

#### Hypotheses tests

The Person-Situation Interaction Hypothesis predicts an IER x Behaviour interaction, whereas the Person Hypothesis predicts an IER main effect on sympathy and supportiveness. We regressed sympathy and supportiveness on behaviour (contrast coded; -1 = control, 1 = undesired behaviour), IER (z-standardized), and the Behaviour x IER interaction. Recalling undesired behaviour significantly reduced sympathy and supportiveness. IER did not moderate this effect, but those who reported more IER also reported more sympathy and supportiveness towards older persons (for statistics, see Table 8). Thus, the present findings again supported the Person but not the Person-Situation Interaction Hypothesis.

**Table 8:** Results of regression analyses, Study 4, *N* = 374.

Criterion	Predictor	*B*	*SE*	β	*t* *	*p*	*CI*_*95%*_ *(B)*	
							*LL*	*UL*
Sympathy	IER	0.26	0.06	0.21	4.17	< .001	0.14	0.38
	Behaviour	-0.16	0.06	-0.13	-2.63	.009	-0.29	-0.04
	IER x Behaviour	-0.004	0.06	-0.003	-0.06	.951	-0.13	0.12
Supportiveness	IER	0.21	0.05	0.22	4.32	< .001	0.11	0.30
	Behaviour	-0.19	0.05	-0.20	-3.95	< .001	-0.28	-0.09
	IER x Behaviour	0.01	0.05	0.01	0.25	.803	-0.08	0.11

LL = Lower Limit, UL = Upper Limit.

*df for main effects: 371; df for interaction: 370.

The present findings also again supported our mediation hypothesis. Following the same procedure as in Studies 1 and 2, we found a significant indirect effect of IER (z-stand.) on supportiveness via sympathy, *B* = 0.10, *SE*_*Boot*_ = 0.03, *CI*_*95%*_[0.05, 0.16].

### Discussion

This study replicated the previous studies using a different manipulation of outgroup behaviour to ensure the behaviour’s personal relevance to participants. The effects of our behaviour manipulation on reported anger, sympathy, and supportiveness confirm that we successfully created personal relevance. Thus, the present results suggest that the lack of support for the Person-Situation Interaction Hypothesis is not due to low relevance of our manipulation. Rather, IER might simply not moderate the effect of behaviour on sympathy and supportiveness but might colour people’s general outlook on outgroups instead. We ran an internal meta-analysis to better judge how substantiated this interpretation is and how strong the predicted effects may be in the population. Moreover, as our Person Hypothesis could be construed as implying a null effect of the IER x Behaviour interaction, we supplemented this meta-analysis with a Bayes Factor analysis based on the meta-analytical effects.

## Meta-analytical summary of the present findings

We ran an internal random-effects meta-analysis with the R-package "metafor" [52]. Thereby, we sought to obtain a better estimate of the true population effects. Moreover, it allows us to better judge whether IER truly does not moderate the effect of behaviour. To base this analysis on all data we had collected on our primary research question before we started writing this manuscript, we also included the data from our preliminary study (see S1 File). We transformed all regression weights (i.e., all *B*s) into z-values and then into correlation coefficients *r*, which we entered into metafor, using the appropriate settings to obtain unbiased estimates for correlations and sampling variances. In case of Study 1, we used the regression weight of the desirability contrast x IER interaction when testing the Person-Situation Interaction Hypothesis.

### Meta-analytical interaction effect as suggested by the Person-Situation Interaction Hypothesis

**Fig 1:**
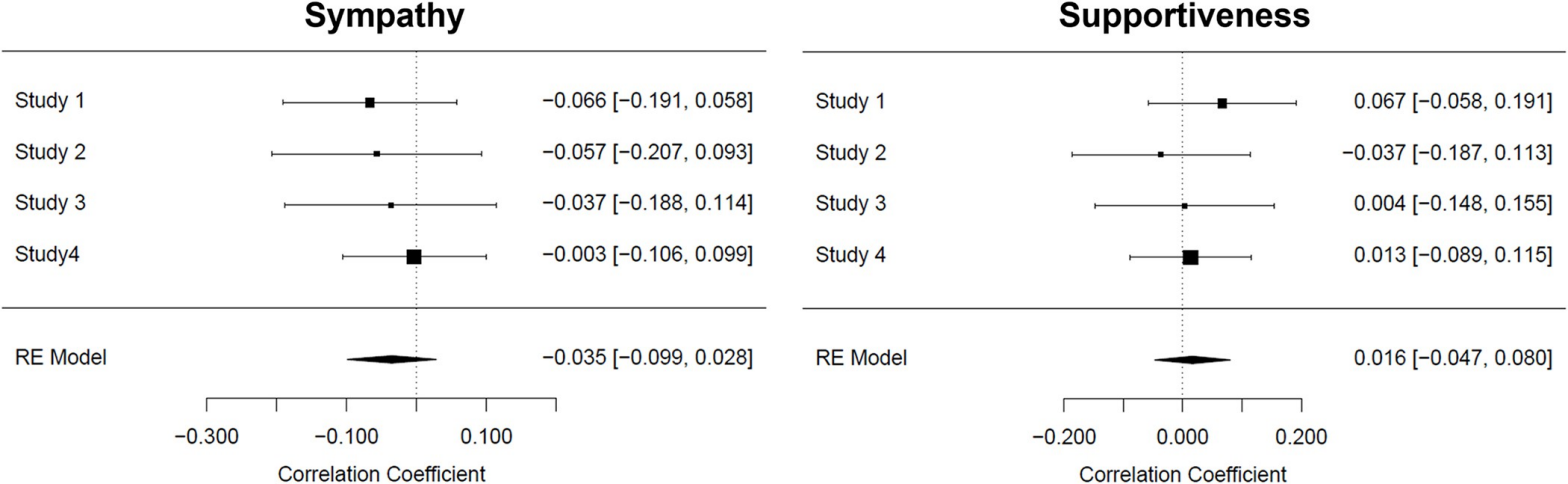
Meta-analysis of the IER x Behaviour interaction effect on sympathy (left) and supportiveness (right), overall *N* = 984. For each study, we display the point estimate for the focal effect (centre of each square), supplemented by a 95% confidence interval calculated around it. The respective values are given on the right-hand side of the graphic. Square size reflects the respective estimate’s weight in computing the average effect based on a study’s sample size. The diamond reflects the average effect size and its 95% confidence interval; the dashed, vertical line reflects the point on the x-axis indicative of a null effect.

For sympathy (see Fig 1, left), the meta-analytical effect size of the IER x Behaviour interaction (-1 = control, 1 = undesired behaviour) was *r* = -0.04, *SE* = 0.03, *z* = -1.09, *p* = .277, *CI*_*95%*_[-0.10, 0.03]. For supportiveness (see Fig 1, right), the IER x Behaviour interaction effect was equally negligible, *r* = 0.02, *SE* = 0.03, *z* = 0.51, *p* = .613, *CI*_*95%*_ [-0.05, 0.08]. Bayes Factor analyses, using the procedure and code proposed by Wetzels and Wagenmakers [53] to compute BF10 suggested that our data provide very strong evidence that there is no IER x Behaviour interaction (BF_10 sympathy_ = 0.05; BF_10support_ = 0.03). Thus, our meta-analysis and the BF analysis both suggest that there likely is no IER x Behaviour effect on either supportiveness or sympathy.

### Meta-analytical IER main effect as suggested by the Person Hypothesis

**Fig 2:**
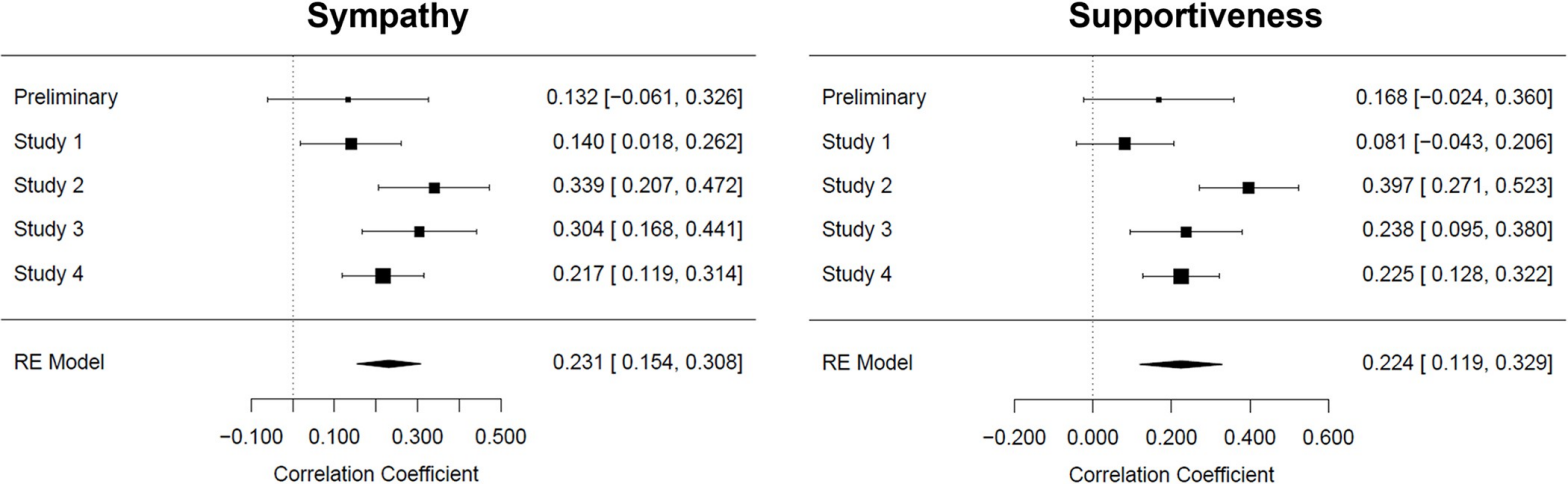
Meta-analysis of the IER main effect on sympathy (left) and supportiveness (right), overall *N* = 1090. For each study, we display the point estimate for the focal effect (centre of each square), supplemented by a 95% confidence interval calculated around it. The respective values are given on the right-hand side of the graphic. Square size reflects the respective estimate’s weight in computing the average effect based on a study’s sample size. The diamond reflects the average effect size and its 95% confidence interval; the dashed, vertical line reflects the point on the x-axis indicative of a null effect.

The meta-analytical main effect of IER on sympathy (see Fig 2, left) was in the small-to-medium range, *r* = 0.23, *SE* = 0.04, *z* = 5.89, *p* < .001, *CI*_*95%*_ [0.15, 0.31]. The meta-analytical main effect of IER on supportiveness (see Fig 2, right) was likewise in this range, *r* = 0.22, *SE* = 0.05, *z* = 4.19 *p* < .001, *CI*_*95%*_ [0.12, 0.33]. Thus, despite the heterogeneity in the individual studies, there likely are small-to-medium, positive IER-sympathy and IER-supportiveness relationships on a population level. Bayes Factor analyses corroborate this interpretation and suggest that our data provide decisive evidence in favour of the Person Hypothesis (BF_10 sympathy_ > 100; BF_10 supportiveness_ > 100).

## General discussion

Negative emotions can prolong and exacerbate intergroup conflicts [e.g., 8,10,11]. Consequently, recent research has increasingly focused on how emotion regulation might improve intergroup relations by reducing negative emotions like anger and fostering positive emotions like hope and sympathy (for a review, see, e.g., [15]). One emotion regulation strategy positively related to outgroup-directed sympathy and, in turn, supportiveness is IER [4]. We set out to test how generalisable this relation is and how it can be explained as this was, to date, unclear.

Across four studies using different methods and outgroups, we consistently found a positive relation between interindividual differences in IER and outgroup-directed supportiveness, mediated via sympathy for the outgroup in question. These findings align with previous research by Roth and colleagues [4], point to their generalizability, and extend them in two meaningful ways. First, where prior research assessed trait sympathy [4], we showed that IER also relates to sympathy for—and consequently supportiveness towards—specific outgroups. Second, the present research provides insights into two possible explanations for this positive relationship.

The first explanation is that interindividual differences in IER shape individuals’ reactions to a concrete situation. IER might mitigate the negative effects undesired (vs not undesired) outgroup behaviour (likely causing negative emotions, e.g., [54]) has on sympathy and supportiveness towards this outgroup. Specifically, people high in IER should strive to understand their emotions’ sources and, thus, be less likely to fall prey to the automatic action tendencies associated with their emotions that would normally prevent them from experiencing sympathy (e.g., [7]). Hence, while facing undesired behaviour by outgroup members should lead to low sympathy for and supportiveness towards this outgroup, this effect should be weaker the higher a person is in IER (i.e., IER should interact with outgroup behaviour; *Person-Situation Interaction Hypothesis*). However, the present research does not support this hypothesis. One might suspect that this lack of evidence is due to low relevance of our materials to participants and, thus, the absence of emotions that required regulation. Given that we found strong effects on reported situative emotions in Studies 1 and 3 and even used a recall of self-relevant situations in Study 4, however, this explanation seems unlikely. Considering the size of the effect estimates from the meta-analysis and the results of the BF analysis, this lack of support for the Person-Situation Interaction Hypothesis also is clearly not due to limited statistical power but rather to there being no relevant effect in the current heterogeneous set of studies.

Instead, our research suggests that the IER—supportiveness relation can be explained by IER colouring people’s general outlook on the outgroup or intergroup relations. IER entails (consistently) attending to one’s own emotions, which might make it more likely that attention is also (consistently) devoted to others’ emotions (Person Hypothesis; 21). In line with this idea, our studies showed a positive relationship between interindividual differences in IER and sympathy as well as supportiveness towards an outgroup, regardless of the situation at hand.

We deem it a major strength of the present research that we found consistent evidence of this relationship using very different target outgroups. Regardless of the outgroup participants faced in the present research, the higher they were in IER, the more sympathy for and supportiveness towards this outgroup they expressed. This points to the generalizability of our findings. Moreover, it suggests that, next to reappraisal [e.g., 2], IER should also be considered as a means of fostering intergroup reconciliation. After all, sympathy with the outgroup has been argued to be an important driver of intergroup forgiveness and support for reconciliatory policies [e.g., 7,15]. Naturally, this requires effective IER-trainings, which might be difficult to implement considering that we did not find IER to affect reactions to situations eliciting negative emotions. Research suggests that changes in child-rearing practices and parents’ socialisation strategies might be needed [e.g., 4,25] to foster IER. Using IER to improve intergroup relations, thus, seems to require a long-term approach.

Nonetheless, a second strength of our research is that we found a positive relationship between interindividual differences in IER and sympathy as well as supportiveness independent of the methodology used. Whereas Study 1 used short clips from news broadcasts, Studies 2 and 3 used vignettes describing relatively commonplace occurrences and observations, and Study 4 used a guided recall procedure. This again speaks in favour of the present findings’ generalizability. Moreover, it suggests that IER might not only be related to more positive intergroup behaviour in exceptional situations like the so-called refugee crisis or violent conflicts such as the conflict between Israel and Palestine but also in everyday intergroup interactions.

Finally, the statistical power of our studies warrants discussion. Given that the power analyses we used to determine sample size a priori did not fully reflect the analyses we interpreted herein, we used two approaches to address power. First, we used sensitivity analyses, which suggested that our samples were large enough to detect effects between *r* = 0.14 and *r* = 0.22 with 80% power. Comparing empirical effect sizes with the smallest detectable effect size reveals that only Study 1 was underpowered to detect the IER-main effect, while the other studies were adequately powered. Second, as all studies individually were underpowered to detect an IER x Behaviour interaction, we conducted an internal meta-analysis and Bayes Factor analyses. Both show that our studies did not fail to show this effect due to a lack of power. Rather, they imply that a Person x Situation interaction is unlikely to underly the link between interindividual differences in IER and sympathy as well as supportiveness. In sum, our studies appear to have been adequately powered to detect the main effect (that, according to the Bayes Factor analysis, likely exists). At the same time, our results suggest that powering a study to detect an IER x Behaviour interaction will require very large samples to have a chance of detecting an effect of negligible practical relevance (that, according to the Bayes Factor analysis, is unlikely to exist).

Next to power, there are three further caveats to the present findings that open avenues for future research. First, although we use the labels "main effect" and "mediation", we use them as purely statistical terms. We cannot make any definitive claims concerning causality in the relation between IER and sympathy or supportiveness from the current studies. This is because we assessed trait-like interindividual differences in IER. Based on prior studies experimentally manipulating sympathy [e.g., 16,17], we would argue that reverse causation (i.e., greater sympathy for the outgroup causing IER or greater supportiveness enhancing sympathy) is unlikely. Nonetheless, we cannot rule out effects of potential confounding variables in the present research. Future research investigating the benefits of IER, thus, should follow the rigorous experimental causal chain design [55]. This requires experimentally inducing IER and testing the effects of this manipulation on sympathy (but see above for potential difficulties). In a second step, sympathy could be manipulated to test whether increases in this variable are indeed the reason why IER is positively related to supportiveness.

That we assessed (trait-like) interindividual differences in IER is also tied to the second caveat that needs to be considered when interpreting the present findings. While we find a consistent positive relationship between IER and prosocial responding, the process underlying this relationship remains unclear. In our theoretical introduction, we proposed that IER may be associated with a stable emotional sentiment characterised by sympathy. Halperin and Pliskin [30] argue that such sentiments emerge when patterns of emotional reactions become ingrained in an individual’s behaviour due to the same target repeatedly eliciting the same emotions, which, in turn, repeatedly cause the same response. Those high in IER should repeatedly experience sympathy (for outgroups) because they allow themselves to experience also potentially unpleasant emotions such as sorrow and concern for others and might be more motivated to take others’ perspectives [24]. By exploring their emotions’ source, they should, in turn, become more likely to realise that outgroup members’ plight causes their emotions and that they value others’ welfare. For this process to occur, however, IER needs to (at least initially) be used in interactions with outgroup members, for which we found no support in the present research. The "book-keeping model" of stereotype change [56] implies that this might be because we used outgroups for which pre-defined stereotypes and attitudes already existed. Hence, future research might want to use outgroups towards which no prior attitudes exist to put our two hypotheses to an even more rigorous test. Alternatively, it might prove insightful to confront participants repeatedly with (not) undesired behaviour by the same group and test whether changes in their responses over time depend on IER. Thereby, we could further improve our understanding of the role IER may play in fostering positive intergroup relations.

Lastly, our focus on intergroup conflicts led us to focus exclusively on how IER relates to sympathy and supportiveness for outgroup members. Viewed against the backdrop of a substantial body of research showing a so-called sympathy-gap (i.e., individuals reporting greater sympathy for ingroup than outgroup members; for reviews, see [17,32]), this raises the question: Could IER reduce this gap? We propose that the answer is likely to be “yes”. This proposition rests on the robust, positive relationship between interindividual differences in IER and outgroup directed sympathy we found herein and on the idea that directing would-be sympathizers’ attention to mental states of outgroup members might help reduce intergroup sympathy biases [57]. However, given that we did not assess sympathy for the ingroup in our studies, future research is needed to test our proposition empirically.

To conclude, the present work aligns with the growing body of research focused on emotion regulation in the context of intergroup conflicts (for an overview, see [15]) and extends it in three meaningful ways. First, it focuses on an emotion regulation strategy that has, to date, rarely been investigated in intergroup conflicts: integrative emotion regulation. Second, it shows that this strategy is not only related to generalised sympathy, as previous work suggests [4] but also to sympathy (and consequently supportiveness) towards specific outgroups. Third, it sheds light on two previously untested possible explanations for the positive relation between IER and outgroup-directed sympathy and supportiveness. The present findings suggest that this positive relationship is not due to IER shaping responses to outgroups in a concrete situation. Rather, it seems to reflect a general tendency to respond with supportiveness and sympathy among those high in IER. Thereby, we address the prominent question of how interindividual differences and situations jointly shape a person’s behaviour.

## Supporting information

S1 File Results of additional analyses. Reports findings of pre-registered analyses (if not already included in main manuscript) and analyses based on the full samples.
(DOCX)
