# Supplementary material for: Integrative emotion regulation relates to sympathy and support for outgroups—Independent of situational outgroup behaviour
Source: PLoS One. 2024 Jan 5;19(1):e0296520. doi: 10.1371/journal.pone.0296520 (PMC10769030; doi:10.1371/journal.pone.0296520)
Supplement: S1 File — Reports findings of pre-registered analyses (if not already included in main manuscript) and analyses based on the full samples. (DOCX) [file pone.0296520.s001.docx]

**SUPPLEMENT S1**

**Contents**

[Preliminary Study 3](#_Toc149569738)

[Deviations from the preregistration 3](#_Toc149569739)

[Method 4](#_Toc149569740)

[Participants & Design 4](#_Toc149569741)

[Procedure 4](#_Toc149569742)

[Measures 5](#_Toc149569743)

[Results 6](#_Toc149569744)

[Elicited Emotions 6](#_Toc149569745)

[Hypotheses tests 6](#_Toc149569746)

[Analyses with full sample 7](#_Toc149569747)

[Discussion 8](#_Toc149569748)

[Pilot Study: Films for Studies 1A/B 9](#_Toc149569749)

[Method 9](#_Toc149569750)

[Participants & Design 9](#_Toc149569751)

[Procedure 9](#_Toc149569752)

[Measures 10](#_Toc149569753)

[Results 10](#_Toc149569754)

[Study 1 A/B 11](#_Toc149569755)

[Deviations from the preregistration 11](#_Toc149569756)

[Results of analyses with the full sample 12](#_Toc149569757)

[Elicited emotions 12](#_Toc149569758)

[Hypotheses tests 12](#_Toc149569759)

[Study 2 13](#_Toc149569760)

[Deviations from the preregistration 13](#_Toc149569761)

[Results of preregistered analyses 13](#_Toc149569762)

[Results of analyses with the full sample 14](#_Toc149569763)

[Exploratory Analyses: Helping an older lady 15](#_Toc149569764)

[Study 3 15](#_Toc149569765)

[Deviations from the preregistration 15](#_Toc149569766)

[Conceptual model of cross-level analyses 16](#_Toc149569767)

[Results of analyses with the full sample 17](#_Toc149569768)

[Elicited Emotions 17](#_Toc149569769)

[Hypotheses tests 17](#_Toc149569770)

[Effect of material set 18](#_Toc149569771)

[Elicited emotions 18](#_Toc149569772)

[Hypotheses tests 18](#_Toc149569773)

[Study 4 19](#_Toc149569774)

[Situating IER in a broader conceptual context 19](#_Toc149569775)

[Deviations from the preregistration 20](#_Toc149569776)

[Method 20](#_Toc149569777)

[Measures 20](#_Toc149569778)

[Controlling for Mindfulness and the Big5 22](#_Toc149569779)

[Results of analyses with full sample 22](#_Toc149569780)

[Elicited Emotions 22](#_Toc149569781)

[Hypotheses Tests 23](#_Toc149569782)

[Controlling for Mindfulness and the Big5 23](#_Toc149569783)

[Discussion 24](#_Toc149569784)

[References 24](#_Toc149569785)

Preliminary Study

This study represents a first attempt at replicating the relation between IER and sympathy as well as supportiveness towards outgroup members reported by Roth and colleagues [1]. Below, we test whether the data support the Person-Situation interaction or the Person Hypothesis. Based on the Person Hypothesis, we would expect a simple main effect of IER: People higher in IER would report higher levels of sympathy and supportiveness towards outgroup members. Based on the Person-Situation interaction Hypothesis, on the other hand, we would expect an interaction between the outgroup's behaviour portrayed in a film clip and IER on sympathy and supportiveness: The effect of the films would be weaker the higher participants are in IER. In this study, we used two types of behaviour, one assumed to elicit fear (information about terroristic threats posed by refugees) and one assumed not to elicit negative emotions (information on identity fraud committed by refugees).

Given that in both cases, undesired behaviour was described and that both clips elicited negative emotions (see below), this study did not allow for a good test of our Person-Situation interaction Hypothesis. Therefore, we decided not to include it in our main manuscript. As this study did allow for a test of our Person Hypothesis, we decided to present it in this supplement instead.

Deviations from the preregistration

We only preregistered the Person-Situation interaction Hypothesis for this study (for preregistration, see <https://aspredicted.org/96v3e.pdf>), which could not sensibly be tested. However, in our analysis plan, we had already included the main effect of IER as a predictor for sympathy and supportiveness. This means that even though we did not preregister it as a hypothesis, the planned analyses allowed drawing conclusions about the Person Hypothesis as well.

Regarding the exclusion of participants, we did not preregister excluding outliers. Not excluding the outlier does not change the pattern of results for sympathy, but the main effect of IER on supportiveness becomes significant, *B* = 0.23, *SE* = 0.10, β = 0.23, *t*(103) = 2.31, *p*= .023, *CI_95%_*[0.03, 0.44] (i.e., more in line with the hypothesis).

Method

Participants & Design

The sample size for this study was determined before data collection with G*Power (Faul et al., 2007), assuming a medium effect (*f*^2^ = 0.15) in a linear multiple regression with three predictors (two main effects, one interaction), an alpha error level of 5% and aiming for 95% power. According to these criteria, 119 participants needed to be collected.

We recruited 123 students from a German university who were randomly assigned to one of the two conditions. In the *terror* condition, they saw a film linking terrorism to refugees. In the *fraud* condition, they saw a film on identity fraud committed by refugees. One participant withdrew their data after the debriefing; another participant had to be excluded from analyses because they showed suspicious answering behaviour (i.e., he/she only ticked the minimum, maximum, or middle value on all items). Moreover, we excluded all participants from the analyses who indicated that German was not their native language (*n* = 14) because we were specifically interested in the relation between Germans and refugees, which might be different for participants with a migration background. Outliers were identified using studentized deleted residual values in a regression with supportiveness as the dependent variable and IER, behaviour, and the Behaviour x IER interaction as predictors. All observations that had a value larger than |2.65| (i.e., were less than about 1% likely to be valid data points) were deemed outliers. One outlier was detected, leaving a sample of 106 participants (*M*_age_ = 23.45, 18-32, 22 males, 83 females, one other) for analysis.

Procedure

Participants completed the whole computerized study set in private cubicles. The current study ran second in a set of three independent studies, but none of the focal effects reported below was moderated by the previous manipulation. After participants had completed the first study of this set, the current study was started. First, we assessed IER and other interindividual differences not relevant to the current research question. Then, participants saw either the terror clip or the fraud clip on the whole screen. Both clips lasted roughly 3.5 minutes.

The *fraud* clip reported on cases of identity fraud committed by refugees to obtain several social welfare payments per month, despite only being entitled to one such payment. The *terror* clip was a combination of two news clips taken from the internet and parts of a documentary that had aired on German national television on terroristic threats linked to refugees.

After participants had watched the respective film clips, we assessed their emotions, including their sympathy towards refugees. Then, we assessed supportiveness by asking participants how much they agreed with different political measures aimed at giving refugees access to the German labor market as well as to the healthcare and educational system. Finally, we measured participants' motivation to behave unprejudiced, their political attitude, suspicions about the study's content, and whether they already knew the film clips. Subsequently, an independent third study was started, after which demographic information was collected. For participation in the whole study set, participants received 8€.

***Measures***

If not indicated otherwise, all variables were assessed on seven-point scales ranging from 1 (don't agree) to 7 (agree).

**IER**. IER was assessed with a German translation of the scale developed by Roth and colleagues [2]. Six items (α = .81), e.g., "When I feel tense or anxious, I try to find out what this tells me about myself and the situation I am in." were used.

**Elicited Emotions**. In total, 17 items assessed participants' emotions after they had seen the film clip on a scale from 1 (not at all) to 7 (very). Four items (i.e., furious, upset, irritated, angry; α = .85) assessed the amount of anger participants felt. Four additional items (i.e., concerned, anxious, uncertain, worried, α = .85) assessed the amount of anxiety participants experienced. For the sake of consistency with the remaining studies, we focused on the two emotion terms "angry" and "anxious" to assess whether the films elicited the intended emotions.

**Sympathy**. Six items (α = .94) measured to what extent participants felt sympathy towards refugees, e.g., "When I think about refugees in general, I am often compassionate". Four of these items were modelled after Batson [3]; two items were modelled after de Vos and colleagues [4].

**Supportiveness**. We used a self-developed scale to assess how strongly participants were in favour of various policies benefitting refugees (nine items, α = .82), e.g., "Refugees should be granted full access to the German health care system", "I think that renting as many cheap flats as possible to house refugees is justified", "The preconditions for getting a permanent residence permit in Germany should be aggravated" (inverse item).

Results

Elicited Emotions

To test whether the terror clip elicited more anxiety than the fraud clip, which in turn was originally assumed not to elicit strong negative emotions, we ran an independent samples t-test on the items “anxious” and “angry”. The terror clip elicited significantly more *anxiety* (*M*= 3.37, *SD* = 1.82) than the fraud clip (*M* = 2.18, *SD* = 1.42, *t* (94.29) = -3.74, *p* < .001). In contrast to our previous assumption, the fraud clip elicited a substantial amount of *anger* (*M* = 4.80, *SD* = 1.73), which was significantly larger than the amount of anger elicited by the terror clip (*M* = 4.10, *SD* = 1.72, *t* (104) = 2.09, *p* = .039). Taken together, this pattern of results suggests that we failed to include a condition in this study that did not involve undesired behaviour. Consequently, this study did not allow for an appropriate test of our Person-Situation Interaction Hypothesis.

Hypotheses tests

To test the assumptions that IER is positively related to sympathy because it (a) reduces the detrimental impact of situations involving undesired behaviour by an outgroup on sympathy and supportiveness or (b) because higher in IER generally react more sympathetic and supportive to outgroups, we ran regression analyses with behaviour (fraud = -1, terror = 1), IER (z-standardized), and the IER x Behaviour interaction.

For *sympathy*, we found no effect of behaviour, *B* = -0.01, *SE* = 0.12, β = -0.01, *t*(103) = -0.12, *p* = .907, CI_95%_[ -0.25, 0.23], no effect of IER, *B* = 0.16, *SE* = 0.12, β = 0.13, *t*(103) = 1.34, *p* = .184, CI_95%_[ -0.08, 0.40], and no IER x Behaviour interaction, *B* = 0.11, *SE* = 0.12, β = 0.09, *t*(102) = 0.88, *p* = .384, CI_95%_[ -0.14, 0.36]. Hence, the present results support neither of our hypotheses.

For *supportiveness*, we likewise found no effect of Behaviour (*B* = 0.13, *SE* = 0.09, β = 0.13, *t*(103) = 1.34, *p* = .183, CI_95%_[ -0.06, 0.31]), no main effect of IER (*B* = 0.16, *SE* = 0.09, β = 0.16, *t*(103) = 1.70, *p* = .093, CI_95%_[ -0.03, 0.35]), and no IER x Behaviour interaction (*B* = -0.01, *SE* = 0.10, β = -0.01, *t*(102) = -0.09, *p* = .925, CI_95%_[ -0.20, 0.18]). Taken together, we also find no support for either of our hypotheses regarding supportiveness towards outgroup members.

Finally, we tested whether IER was indirectly related to supportiveness towards outgroup members via enhanced sympathy using PROCESS (v 2.16.3; [5]; model 4) and treated IER (z-standardized) as the predictor, sympathy as the mediator, and supportiveness as the outcome variable. However, we found no indirect effect via sympathy, *B*= 0.07, *SE_Boot_* = 0.05, *CI_95%_*[ -0.02, 0.19]. Hence, the present findings do not replicate the work by Roth and colleagues (Roth et al., 2017).

Analyses with full sample

As the last step, we tested whether excluding participants had unduly influenced our key findings. To this end, we re-ran all analyses based on the full sample (*N* = 122). The results of these analyses support our Person Hypothesis.

For *sympathy*, we found no effect of behaviour (*B* = -0.03, *SE* = 0.11, β = -0.02, *t*(119) = -0.25, *p* = .802, CI_95%_[ -0.24, 0.19]), but a marginal effect of IER (*B* = 0.21, *SE* = 0.11, β = 0.17, *t*(119) = 1.88, *p* = .063, CI_95%_[ -0.01, 0.42]). The IER x Behaviour interaction was, however, not significant (*B* = 0.02, *SE* = 0.11, β = 0.02, *t*(118) = 0.21, *p* = .838, CI_95%_[ -0.20, 0.25]).

For *supportiveness*, there was no effect of behaviour (*B* = 0.11, *SE* = 0.09, β = 0.11, *t*(119) = 1.23, *p* = .220, CI_95%_[ -0.07, 0.28]), but a significant effect of IER (*B* = 0.21, *SE* = 0.09, β = 0.21, *t*(119) = 2.31, *p* = .022, CI_95%_[ 0.03, 0.38]). The IER x Behaviour interaction was, however, not significant, *B* = -0.06, *SE* = 0.09, β = -0.06, *t*(118) = -0.64, *p* = .522, CI_95%_[ -0.24, 0.12]. Thus, analyses with the full sample support our Person Hypothesis concerning supportiveness, as they suggest that the higher participants were in IER, the more supportiveness towards refugees they expressed.

Finally, we tested whether the positive relation between IER and supportiveness was mediated by increased sympathy. In line with our prediction, the indirect effect was significant, *B*= 0.09, *SE_Boot_* = 0.06, *CI_95%_*[ 0.001, 0.22].

Discussion

Taken together, this study provided support for neither of our hypotheses when our preregistered exclusion criteria were applied. Analyses with the full sample, however, speak in favour of our Person Hypothesis, such that the higher participants were in IER, the more supportive they were towards an outgroup, mediated via higher sympathy. Given that the effects of IER were, however, not significant in the reduced sample, this should not be overinterpreted. We assume that two issues prevented us from finding support for our hypotheses.

First, our study lacked a neutral control condition. Therefore, it does not allow drawing firm conclusions regarding our Person-Situation interaction Hypothesis, as this hypothesis predicts that IER mitigates the adverse effects of an outgroup's undesired behaviour on sympathy for and supportiveness towards this group. Second, this study might have failed to produce evidence in favour of our Person Hypothesis due to the relatively small size of the reduced sample.

Pilot Study: Films for Studies 1A/B

Based on the results of our preliminary study concerning the emotions elicited by the film clips, we decided to expand the two already used clips and to search for an additional film clip not portraying undesired behaviour (i.e., a clip not eliciting negative emotions). In total, we devised a set of three clips - a fraud clip, a terror clip, and a control clip. These were then pilot tested to investigate whether they would elicit specific negative emotions. We assumed that the fraud clip would elicit more anger than the remaining two clips and that the terror clip would elicit more anxiety than the remaining two clips.

Method

Participants & Design

For this study, we recruited 45 participants (*M*_age_ = 22.31, 19-32, 13 males, 30 females, 1 other, 1 participant did not indicate their gender) in the foyer of the university's library. Participants were randomly assigned to view one of the three film clips: fraud, terror, or control.

Procedure

Participants were seated at individual laptops. After they had provided informed consent to participate, we showed them one of three film clips, two of which were focused on undesired behaviour by refugees. In the “*undesired behaviour - fraud*” condition, participants viewed a clip detailing cases of identity fraud committed by refugees to obtain several social welfare payments and violent outbreaks in a housing facility for refugees attributed to a lack of wireless internet. In the “*undesired behaviour - terror*” condition, participants viewed a clip describing terroristic attacks in Germany and neighbouring countries committed by refugees and reporting that ISIS might have smuggled terrorists into Europe disguised as refugees. In the “*no undesired behaviour*” control condition, participants received information about prejudice against refugees and accusations levelled at them as well as information on why these are false.

Subsequently, we assessed participants' emotions and how credible they found the film clip. This was followed by demographic questions, questions assessing contact with refugees, and a measure of participants' political attitudes.

Measures

In total, we used 16 items to assess how strongly participants experienced several emotions on a scale from 1 (not at all) to 7 (very). Four items (concerned, anxious, uncertain, worried; α = .83) assessed anxiety. Four items (furious, upset, irritated, angry; α = .83) assessed anger. To remain consistent with the main manuscript, we focus on the two items “anxious” and “angry” in the analyses reported below. The remaining eight items assessed positive emotions and served as distractors.

Results

To test whether the pattern of emotions elicited by our film clips was as expected, we ran two ANOVAs with condition as the between participants factor and anger and anxiety, respectively, as outcomes. For elicited *anger*, we found a significant effect of condition, *F*(2,42) = 5.00, *p* = .011, η_p_^2^ = .192. Simple comparisons suggest that the fraud clip (*M* = 4.27, *SD* = 1.67) elicited significantly more anger than the control clip (*M* = 2.47, *SD* = 1.19, *p* = .003). Moreover, it elicited marginally more anger than the terror clip (*M* = 3.13, *SD* = 1.81, *p* = .056). The amount of elicited anger did not differ between the control clip and the terror clip, *p* = .253.

For elicited *anxiety*, we likewise found a significant effect of condition, *F* (2,42) = 4.80, *p* = .013, η_p_^2^ = .186, such that the terror-clip (*M* = 2.93, *SD* = 1.58) elicited significantly more anxiety than the neutral clip (*M* = 1.53, *SD* = 0.92, *p* = .004) and marginally more anxiety than the terror clip (*M* = 2.07, *SD* = 1.16, *p* = .064). The amount of anxiety elicited by the control clip and the fraud clip did not differ, *p* = .249.

In sum, this pilot study suggests that the clips used in the undesired behaviour conditions indeed elicited negative emotions. Moreover, these emotions differed according to the respective film used. The fraud clip primarily elicited anger, whereas the terror clip primarily elicited anxiety. Compared to these two clips, the neutral control clip did not elicit high amounts of either negative emotion, which led us to use it as a control condition in Study 1A/B.

Study 1 A/B

Deviations from the preregistration

When preregistering Study 1A, we were optimistic that we would succeed in collecting the required sample size in a single study. Therefore, we did not preregister that we would do another round of data collection later. Unfortunately, we failed to collect the number of valid responses suggested by our a priori power analysis in Study 1A. Thus, we decided to run the same study again (using the same preregistration) and to combine the two data sets. This led us to exclude more participants from the sample than we had originally planned. Analyses with the full sample are reported below.

For this study, we again only explicitly preregistered the Person-Situation interaction Hypothesis. However, the main effect of IER postulated in the Person Hypothesis again was part of our analysis plan, meaning that the study allows drawing conclusions about this effect. Regarding planned analyses, we preregistered testing a moderated mediation in which IER should moderate the link between behaviour and sympathy, which should in turn predict supportiveness. However, given the lack of a Behaviour x IER interaction on both sympathy and supportiveness, we opted for conducting a regular mediation analysis instead.

Finally, two oversights on our part need to be noted. First, we did not specify the regression that would be used as a criterion for outlier exclusions, yet did specify our planned cut-off value. Second, we ran our a priori power analysis based on seven predictors. Since the two contrasts are orthogonal, any terms involving interactions between the two are obviously zero. This means that we should have planned our sample based on five predictors, which would have resulted in a minimum sample size of *N* = 171.

Results of analyses with the full sample

Elicited emotions

An ANOVA used to test whether participants' emotions were contingent on experimental condition revealed significant effects on both anger, *F* (2,344) = 39.51, *p* < .001, η_p_^2^ = .187, and anxiety, *F* (2,344) = 54.95, *p* < .001, η_p_^2^ = .242. Elicited *anger* was higher in the “undesired behaviour - fraud” condition (*M* = 5.34, *SD* = 1.45) than in both the “undesired behaviour-terror” condition (*M* = 4.65, *SD* = 1.66, *p* = .002) and the control condition (*M* = 3.41, *SD* = 1.89, *p* < .001 ), which in turn also differed in elicited anger, *p* < .001. Elicited *anxiety* was higher in the “undesired behaviour - terror” condition (*M* = 3.46, *SD* = 1.72) than in both the “undesired behaviour-fraud” condition (*M* = 2.73, *SD* = 1.50, *p* < .001) and the control condition (*M* = 1.48, *SD* = 1.03, *p* < .001), which in turn also differed in elicited anxiety, *p* < .001.

Hypotheses tests

A regression analysis with *sympathy* as the dependent variable revealed no effect of the desirability contrast comparing the control condition (coefficient: -2) to the two conditions with undesired behaviour (coefficients: 1), *B* = -0.01, *SE* = 0.05, β = ‑0.01, *t*(343) = ‑0.15, *p*= .879, *CI_95%_*[ -0.10, 0.08]. The effect of the residual contrast (fraud = 1, terror = -1) was marginal, *B* = 0.15, *SE* = 0.08, β = 0.10, *t*(343) = 1.88, *p*= .061, *CI_95%_*[ -0.01, 0.30]. In line with our Person Hypothesis, we found a significant main effect of IER, *B* = 0.15, *SE* = 0.06, β = 0.12, *t*(343) = 2.31, *p*= .022, *CI_95%_*[ 0.02, 0.28], but no evidence of an IER x Behaviour interaction, both |*B*|s < 0.07, both *p*s > .180.

For *supportiveness*, no effect of the two contrasts emerged, both |*B*|s < 0.07, both *p*s > .115. There was a trend for a main effect of IER, *B* = 0.09, *SE* = 0.06, β = 0.09, *t*(343) = 1.65, *p*= .100, *CI_95%_*[ ‑0.02, 0.21], but no evidence of an IER x Behaviour interaction, both |*B*|s < 0.03, both *p*s > .560.

Finally, we again ran a mediation analysis to test whether IER would be indirectly related to supportiveness via sympathy. In line with this idea, we found a significant indirect effect of IER on supportiveness via sympathy, *B* = 0.08, *SE_Boot_* = 0.04, *CI_95%_*[ 0.01, 0.16].

Taken together, these analyses speak in favour of the robustness of the effects reported in the main manuscript, as they did not substantially change when all observations were included.

Study 2

Deviations from the preregistration

This study was originally intended for a different purpose (see preregistration: <https://aspredicted.org/w4bz7.pdf>). Specifically, we intended to test how IER interacts with right-wing authoritarianism (RWA) and social dominance orientation (SDO). Therefore, the results reported for Study 2 are based on a manipulation implemented for exploratory purposes. Given that the present study's results can shed light on the validity of the hypotheses reported in the main manuscript, we nonetheless decided to include the study. Seeing as we employed different predictors in our analyses, we also changed the basis for outlier exclusion from a regression involving RWA, IER and their interaction to one involving Behaviour, IER, and their interaction. As the a priori power analysis is not sensitive to which predictors are used, only to how many, its appropriateness is not affected by our decision to focus on a different aspect of the study.

Results of preregistered analyses

We used the same exclusions for the analyses reported below as we did in the main manuscript. We assessed *RWA* with a questionnaire developed by Beierlein and colleagues [6]. Participants indicated their agreement with nine items (α = 0.80) on a 5-point scale (don't agree at all - agree a little - somewhat agree - rather agree - fully agree). *SDO* was assessed with Cohrs & Asbrock's [7] scale. Participants indicated their agreement with twelve items (α = 0.78) on a scale from 1 (don't agree at all) to 7 (fully agree).

We z-standardized the continuous predictors and entered them simultaneously into a linear regression to test our preregistered hypotheses, adding their interaction in a second step. We found no effects involving RWA for *sympathy* as the criterion, both |*B*|s < 0.13, both *p*s > .150. We also did not find the predicted SDO x IER interaction, *B* = 0.03, *SE* = 0.07, β = 0.03, *t*(174) = 0.38, *p*= .703, *CI_95%_*[-0.10, 0.16], but only a significant main effect of SDO, *B* = ‑0.20, *SE* = 0.08, β = -0.17, *t*(175) = ‑2.41, *p*= .017, *CI_95%_*[-0.36, -0.04]. Hence, the higher participants' social dominance orientation, the less sympathy towards the outgroup they reported.

For *supportiveness* as the criterion, we likewise found no effects involving RWA, both |*B*|s < 0.05, both *p*s > .570. As for sympathy, the predicted SDO x IER interaction did not reach significance, *B* = -0.03, *SE* = 0.06, β = -0.03, *t*(174) = -0.47, *p*= .640, *CI_95%_*[-0.15, 0.09], but a significant SDO main effect emerged, *B* = -0.19, *SE* = 0.08, β = -0.17, *t*(175) = -2.40, *p*= .017, *CI_95%_*[-0.34, -0.03]. Again, the higher participants scored on SDO, the less supportiveness towards the elderly did they report. Due to the absence of significant interactions, we did not test for the preregistered moderated mediation.

Results of analyses with the full sample

For *sympathy*, our regression analysis revealed a marginal effect of behaviour, *B* = ‑0.14, *SE* = 0.08, β = -0.12, *t*(201) = -1.74, *p*= .084, *CI_95%_*[ ‑0.29, 0.02], suggesting that participants reported less sympathy in the undesired behaviour condition than in the control condition. In line with the Person Hypothesis, we found a positive main effect of IER, *B* = 0.36, *SE* = 0.08, β = 0.31, *t*(201) = 4.65, *p*< .001, *CI_95%_*[ 0.21, 0.52]. We found no evidence for an IER x Behaviour interaction, *B* = ‑0.05, *SE* = 0.08, β = -0.04, *t*(200) = -0.58, *p*= .565, *CI_95%_*[ ‑0.20, 0.11], which would have been predicted by our Person-Situation Interaction Hypothesis.

For *supportiveness*, we found no main effect of behaviour, *B* = -0.04, *SE* = 0.08, β = ‑0.04, *t*(201) = -0.56, *p*= .573, *CI_95%_*[ -0.20, 0.11], but a significant main effect of IER, *B* = 0.33, *SE* = 0.08, β = 0.29, *t*(201) = 4.29, *p*< .001, *CI_95%_*[ 0.18, 0.49]. As for sympathy, we found no evidence for an IER x Behaviour interaction, *B* = -0.02, *SE* = 0.08, β = -0.01, *t*(200) = -0.21, *p =* .834, *CI_95%_*[ -0.17, 0.14].

In our mediation analysis, we again found a significant indirect effect of IER on supportiveness via sympathy, *B* = 0.17, *SE_Boot_* = 0.04, *CI_95%_*[ 0.10, 0.27]. This means that for this study, exclusions again did not substantially affect the pattern of results: Even with the full sample, we found no evidence in favour of our Person-Situation Interaction Hypothesis, but rather for our Person Hypothesis.

Exploratory Analyses: Helping an older lady

After assessing sympathy and support in general, we asked our participants to imagine seeing an older lady’s shopping bags tearing a short distance away, spilling her groceries onto the street. Subsequently, we asked participants how likely they would show six different behaviours (e.g., taking their own shopping bags from their car to give to the older lady, offering to drive the old lady home; α = .70). A step-wise regression analysis including IER and behaviour in the first step and their interaction in a second step revealed, consistent with the results for our more general measure of supportiveness, a significant IER main-effect, *B* = 0.33, *SE* = 0.07, β = 0.35, *t*(175) = 5.01, *p*< .001, *CI_95%_*[ 0.20, 0.47]. Outgroup behaviour did not affect participants’ readiness to help in a specific situation, *B* = -0.02, *SE* = 0.07, β = -0.02, *t*(175) = -0.29, *p*= .776, *CI_95%_*[ -0.15, 0.11], nor was there an IER x Behavior interaction, *B* = ‑0.01, *SE* = 0.07, β = -0.01, *t*(174) = -0.07, *p*= .942, *CI_95%_*[ -0.14, 0.13]. Hence, our exploratory analysis suggests that IER might not only foster general but also situation-specific supportiveness for outgroup members. Moreover, this analysis again points to the validity of our Person Hypothesis but is not in line with our Person-Situation-Interaction hypothesis.

Study 3

Deviations from the preregistration

We deviated from our preregistration in the terms we used. In the preregistration, the Person-Situation interaction Hypothesis is called "Situation Hypothesis". Moreover, sympathy is called "empathy", supportiveness is called "policy support", we used the term "norm violations" to denote what in the main article is called undesired behaviour, and refer to behaviour described in the control condition as "normative behaviour". Other than preregistered, we did not control for group as a level 1 variable. To be able to interpret the effects of this variable, it would have been necessary to create 15 dummy variables, each comparing one group to another. Moreover, these comparisons would have been confounded by the compared groups' behaviour. Therefore, we only provide information on the effect of material set below.

Conceptual model of cross-level analyses

To test our mediation hypothesis in Study 3, we followed the structural equation modelling approach suggested by Preacher, Zhang and Zyphur [8]. This means that we first modelled sympathy and supportiveness both on Levels 1 and 2 of our model. Essentially, this means that the variance in these variables is separated into a within-part (representing *intra*individual differences in the variables) and a between-part (representing interindividual differences in the variables). Then, we regressed *intra*individual differences in supportiveness on *intra*individual differences in sympathy. Moreover, we regressed *inter*individual differences in supportiveness on *inter*individual differences in sympathy and regressed *inter*individual differences in both variables on IER.

**Figure S1.**

Conceptual model of multi-level mediation analysis


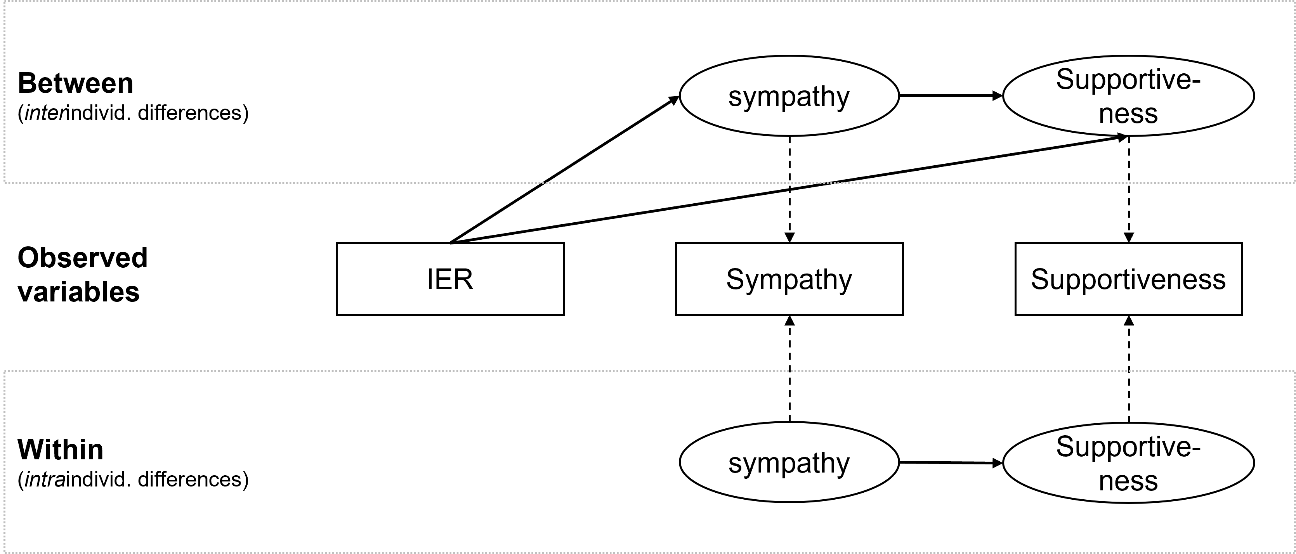


Figure S1 depicts the model underlying this analysis. Solid arrows represent paths estimated in the model, and dashed arrows represent the separation of the variance in sympathy and supportiveness into (latent) within- and between-participant differences. Rectangles represent manifest, observed variables. Note that IER could only be entered as a predictor of the between-person relations because it did not vary within participants.

Results of analyses with the full sample

Elicited Emotions

First, we again tested on the within-participant level whether behaviour (-1 = control, 1 = undesired behaviour) predicted participants' emotions. In line with the findings reported in the main manuscript, we found a significant effect of behaviour on anger, *B* = 1.48, *SE* = 0.05, *z* = 29.94, *p*< .001, *CI_95%_*[ 1.38, 1.58], as well as anxiety, *B* = 0.37, *SE* = 0.03, *z* = 11.03, *p*< .001, *CI_95%_*[ 0.30, 0.43]. Thus, our undesired behaviour manipulation worked as intended.

Hypotheses tests

We used the same multi-level approach as in our main manuscript. For *sympathy*, we found no evidence that IER moderated the strength of the relation between behaviour and sympathy, *B* = -0.04, *SE* = 0.04, *z* = -0.83, *p*= .406, *CI_95%_*[ ‑0.12, 0.05]. However, we found a significant effect of IER on sympathy, *B* = 0.31, *SE* = 0.08, *z* = 4.08, *p*< .001, *CI_95%_*[ 0.16, 0.45]. This implies that people who are higher in IER respond with greater sympathy to outgroups regardless of how members of this group behaved. This pattern is fully in line with the results reported in our main manuscript.

In an analogous analysis for *supportiveness*, we likewise found no evidence of a moderation by IER, *B* = -0.01, *SE* = 0.02, *z* = -0.57, *p*= .569, *CI_95%_*[ -0.06, 0.03], but evidence of an IER main effect, *B* = 0.21, *SE* = 0.07, *z* = 3.26, *p*= .001, *CI_95%_*[ 0.08, 0.34]. This result pattern is, again, fully in line with the findings reported in the main manuscript.

In our mediation analysis, we found a positive relation between sympathy and supportiveness on the within-person level, *B* = 0.55, *SE* = 0.03, *z* = 20.80, *p*< .001, *CI_95%_*[ 0.50, 0.60]. On the between-person level, sympathy was positively related to IER, *B* = 0.31, *SE* = 0.08, *z* = 4.08, *p*< .001, *CI_95%_*[ 0.16, 0.45], and in turn predicted supportiveness, *B* = 0.76, *SE* = 0.08, *z* = 9.06, *p*< .001, *CI_95%_*[ 0.59, 0.92]. IER did not have a direct effect on supportiveness, *B* = -0.02, *SE* = 0.05, *z* = -0.37, *p*= .711, *CI_95%_*[ -0.12, 0.09]. Importantly, however, the indirect effect of IER on supportiveness via sympathy was significant, *B* = 0.23, *SE* = 0.06, *z* = 4.01, *p*< .001, *CI_95%_*[ 0.12, 0.34]. Again, this pattern is fully in line with the findings reported in the main manuscript.

Effect of material set

Elicited emotions

To test whether the material set participants received moderated the effects of behaviour on participants' emotions, we used the same random coefficient logic as for sympathy and supportiveness but employed the MLF-estimator. However, instead of IER, we included material set (coded Set A = 1, Set B = ‑1) as a Level-2 predictor of the random slope of emotion regressed on behaviour. The effect of behaviour on *anger* was moderated by material set, *B* = ‑0.21, *SE* = 0.05, *z* = -4.10, *p*< .001, *CI_95%_*[ -0.31, -0.11], such that the effect of undesired behaviour was larger in Set B, *B* = 1.75, *SE* = 0.09, *z* = 20.48, *p <* .001, *CI_95%_*[ 1.59, 1.92], than in Set A, *B* = 1.34, *SE* = 0.08, *z* = 16.40, *p*< .001, *CI_95%_*[ 1.18, 1.50]. It did, however, not moderate the effect of behaviour on anxiety, *B* = 0.11, *SE* = 0.08, *z* = 1.30, *p*= .194, *CI_95%_*[ ‑0.05, 0.27]. In sum, this suggests that in Material Set B, more anger was elicited by undesired behaviour than in Set A. However, given that both sets still elicited negative emotions, this does not call our manipulation into question.

Hypotheses tests

To test whether material set also plays a role for sympathy and supportiveness, we again included this factor as a Level-2 variable into our analyses. It was included alongside IER and an IER x Set interaction.

For *sympathy*, we found a significant moderation effect of material set on the effect of behaviour on sympathy, *B* = 0.43, *SE* = 0.03, *z*= 14.41, *p*< .001, *CI_95%_*[ 0.37, 0.49]. Undesired behaviour affected sympathy positively in Set A, *B* = 0.37, *SE* = 0.04, *z*= 8.74, *p*< .001 , *CI_95%_*[ 0.29, 0.46], but affected sympathy negatively in Set B, *B* = -0.49, *SE* = 0.04, *z*= ‑11.67, *p*< .001, *CI_95%_*[ -0.57, -0.41]). Importantly, there was no significant IER x Behaviour x Material Set three-way interaction on sympathy, *p* > .750, nor was the IER main effect on sympathy moderated by material set, *p* > .195.

For *supportiveness*, the result pattern was identical. Material set moderated the effect of behaviour on supportiveness, *B* = 0.19, *SE* = 0.05, *z*= 4.14, *p*< .001, *CI_95%_*[ 0.10, 0.28]). Undesired behaviour affected supportiveness positively in Set A, *B* = 0.14, *SE* = 0.06, *z*= 2.12, *p*= .034, *CI_95%_*[ 0.01, 0.26], but was negatively related to supportiveness in Set B, *B* = -0.24, *SE* = 0.07, *z*= -3.69, *p*< .001, *CI_95%_*[ ‑0.37, -0.11]). Again, there was no evidence of a Materials x IER interaction effect on supportiveness, p > .150, nor of an IER x Behaviour x Material Set interaction, *p* > .785.

In our cross-level mediation analysis, we found a positive relation between sympathy and supportiveness on the within-participant level. *B* = 0.55, *SE* = 0.03, *z* = 19.53, *p <* .001, *CI_95%_*[ 0.50, 0.61]. On the between-participant level, we found a positive relation between IER and sympathy, *B* = 0.32, *SE* = 0.08, *z* = 4.21, *p*< .001, *CI_95%_*[ 0.17, 0.47], but no other effect on sympathy was significant, *p*s > .195. Supportiveness was significantly associated with sympathy, *B* = 0.74, *SE* = 0.09, *z* = 8.42, *p*< .001, *CI_95%_*[ 0.57, 0.91], and was marginally affected by material set, *B* = -0.09, *SE* = 0.05, *z* = -1.88, *p*= .060, *CI_95%_*[ -0.18, 0.004].

In combination, these results suggest that, even though individuals react differently to undesired behaviour by different groups, those higher in IER consistently respond with greater sympathy and supportiveness to outgroups. The key findings were not contingent on the set of scenarios participants read.

Study 4

Situating IER in a broader conceptual context

As discussed in the introduction, one might, at first sight, assume that IER is closely related to mindfulness. To test this suspicion and to situate IER in the broader literature on personality, we used a latent structural equation modelling approach as recommended by Westfall and Yarkoni [9]. We ran the analyses using MPlus version 8.1 [10], using the ML estimator and analysis type general. We report fully standardised coefficients (MPlus STDYX). For IER, mindfulness, sympathy, and supportiveness, we used the respective items as indicators for the latent factors. For the Big5, the three sub-facets per dimension assessed in the BFI-2 [11] served as manifest indicators. However, including all Big5 alongside IER and mindfulness does not seem appropriate here.

Agreeableness, as conceptualised by Soto and John [11], contains a compassion subscale. On a theoretical level, this means that agreeableness should capture trait variations in our key dependent variable, sympathy. While we assessed sympathy not as a trait but rather related to a specific outgroup, controlling for agreeableness when trying to position IER in relation to the Big5 and mindfulness nonetheless binds relevant variance in the dependent variable sympathy (and consequently supportiveness).

Deviations from the preregistration

We deviated from the preregistration by changing the labels for our hypotheses from "State" to "Person-Situation interaction" and from "Trait" to "Person". One of the two remaining deviations from the preregistration is mentioned in the main manuscript. That is, we excluded also participants who did not describe a specific situation or any situation at all, as this was required for our manipulation. The analyses reported herein deviate from our pre-registration in a second way: As outlined above, we did not control for agreeableness in the latent SEM due to the strong conceptual overlap between this personality dimension and sympathy, our dependent variable.

Method

Measures

**Trait Mindfulness.** Participants answered the German version of the Mindful Attention and Awareness Scale (MAAS; [12]) comprising 15 items (α=.87) on a scale from 1 ("almost never") to 6 ("almost always"); e.g. "I could be experiencing some emotion and not be conscious of it until some time later". Items were recoded and averaged into an index for which higher scores indicate higher mindfulness.

**Big Five Personality Traits.** We used the German version of the Big Five Inventory 2 (BFI-2; [13]) to capture Openness (α=.68), Conscientiousness (α=.77), Extraversion (α=.76), Agreeableness (α=.72), and Neuroticism (α=.83) on a scale from 1 ("do not agree at all") to 5 ("fully agree"). Each Big5 score (including reliability) was computed based on its three sub-facets, which we assessed with four items each (for sub-facet reliabilities and intercorrelations, see Table S1).

**Table S1.**

Big 5 Facet reliabilities and correlations with facets assessing the same dimension (all correlations significant at *p* < .001)

| Dimension | Facet | α | *r*1 | *r*2 |
| --- | --- | --- | --- | --- |
| Openness | Intellectual Curiosity | .73 | .44 | .50 |
|  | Aesthetic Sensitivity | .88 |  | .35 |
|  | Creative Imagination | .82 |  | - |
| Conscientiousness | Organization | .86 | .56 | .52 |
|  | Productiveness | .76 |  | .57 |
|  | Responsibility | .63 |  | - |
| Extraversion | Sociability | .86 | .58 | .55 |
|  | Assertiveness | .82 |  | .41 |
|  | Energy Level | .70 |  | - |
| Agreeableness | Compassion | .79 | .49 | .48 |
|  | Respectfulness | .67 |  | .44 |
|  | Trust | .71 |  | - |
| Neuroticism | Anxiety | .81 | .66 | .70 |
|  | Depression | .86 |  | .50 |
|  | Emotional Volatility | .79 |  | - |

Controlling for Mindfulness and the Big5

Based on the reasoning provided above, we ran the latent SEMs without agreeableness as a predictor^[[1]](#footnote-1)^. In these analyses, the relations between IER and sympathy (β=0.17, *SE*=0.07, *z=*2.56, *p=*.011, CI_95%_[0.04, 0.30]) and supportiveness (β=0.19, *SE*=0.08, *z=*2.52, *p=*.012, CI_95%_[0.04, 0.34]) are significant.

Results of analyses with full sample

Elicited Emotions

Analyses with the full sample replicate the pattern reported in the main manuscript. An independent samples t-test again showed that participants in the undesired behaviour condition reported significantly more anger (*M*=5.46, *SD*=1.43) than participants in the control condition (*M*=1.57, *SD*=1.28, *t*(432.98)=29.99, *p* < .001).

Hypotheses Tests

The results reported in the main manuscript also replicate with the full sample regarding sympathy and supportiveness. For sympathy, significant main effects of behaviour (*B*=-0.14, *SE*=0.06, β=-0.11, *t*(436)=-2.43, *p=*.015, *CI_95%_*[-0.26, -0.03]) and IER (*B*=0.27, *SE*=0.06, β=0.22, *t*(436)=4.63, *p*< .001, *CI_95%_*[0.16, 0.39]) emerged. The Behaviour x IER interaction effect, however, was not significant, *B*=-0.01, *SE*=0.06, β=-0.01, *t*(435)=-0.17, *p=*.865, *CI_95%_*[‑0.13, 0.11]. For *supportiveness*, the result pattern was identical. The main effects of behaviour (*B*=‑0.17, *SE*=0.05, β=-0.16, *t*(436)=-3.51, *p*< .001, *CI_95%_*[-0.26, -0.07]) and IER (*B*=0.17, *SE*=0.05, β=0.17, *t*(436)=3.57, *p*< .001, *CI_95%_*[0.08, 0.27]) were signficant, but the Behaviour x IER interaction effect was not (*B*=0.01, *SE*=0.05, β=0.01, *t*(435)=0.30, *p=*.767, *CI_95%_*[-0.08, 0.11]). Thus, also analyses based on the full sample only provide evidence for the Person Hypothesis but not the Person-Situation interaction Hypothesis.

Also regarding our mediation hypotheses, findings from the full sample are in line with the analyses in the main manuscript. As reported there, the indirect effect of IER on supportiveness via sympathy was significant, *B*=0.12, *SE_Boot_*=0.03, *CI_95%_*[0.06, 0.18].

Controlling for Mindfulness and the Big5

Also the analyses using SEM yielded the same result pattern as above when the whole sample was used. We found a significant relation between IER and sympathy (β=0.19, *SE*=0.06, *z=*3.22, *p=*.001, CI_95%_[0.07, 0.30]), as well as between IER and support (β=0.15, *SE*=0.07, *z=*2.24, *p=*.025, CI_95%_[0.02, 0.28]) while controlling for mindfulness, openness, conscientiousness, extraversion, and neuroticism.

Discussion

This study provides initial evidence allowing us to position IER in the context of other concepts from research on emotion regulation, specifically mindfulness, and from research on personality, namely the Big5. Our results suggest that IER and mindfulness might appear highly similar at first sight but are only weakly correlated and have different correlates. Further, they suggest that IER explains variance in sympathy and supportiveness that is not accounted for by openness, conscientiousness, extraversion, and neuroticism. Thus, to predict sympathy and supportiveness, it seems worthwhile to focus on the narrow concept of IER in lieu of the Big5 (although our findings do not prove that IER is unrelated to the Big5, see Bainbridge et al., 2022).

References

1. Roth G, Shane N, Kanat-Maymon Y. Empathising with the enemy: emotion regulation and support for humanitarian aid in violent conflicts. Cogn Emot. 2017;31: 1511–1524. doi:10.1080/02699931.2016.1237348

2. Roth G, Assor A, Niemiec CP, Ryan RM, Deci EL. The emotional and academic consequences of parental conditional regard: Comparing conditional positive regard, conditional negative regard, and autonomy support as parenting practices. Dev Psychol. 2009;45: 1119–1142. doi:10.1037/a0015272

3. Batson CD. The altruism question: Toward a social-psychological answer. Hillsdale, NJ, USA: Erlbaum; 1991.

4. de Vos B, van Zomeren M, Gordijn EH, Postmes T. The Communication of “Pure” Group-Based Anger Reduces Tendencies Toward Intergroup Conflict Because It Increases Outgroup Empathy. Pers Soc Psychol Bull. 2013;39: 1043–1052. doi:10.1177/0146167213489140

5. Hayes AF. Introduction to mediation, moderation, and conditional process analysis: a regression-based approach. Second edition. New York: Guilford Press; 2018.

6. Beierlein C, Asbrock F, Kauff M, Schmidt P. Die Kurzskala Autoritarismus (KSA-3): ein ökonomisches Messinstrument zur Erfassung dreier Subdimensionen autoritärer Einstellungen. Mannheim: GESIS - Leibniz-Institut für Sozialwissenschaften; 2014. Available: http://nbn-resolving.de/urn:nbn:de:0168-ssoar-426711

7. Cohrs JC, Asbrock F. Right-wing authoritarianism, social dominance orientation and prejudice against threatening and competitive ethnic groups. Eur J Soc Psychol. 2009;39: 270–289. doi:10.1002/ejsp.545

8. Preacher KJ, Zhang Z, Zyphur MJ. Alternative Methods for Assessing Mediation in Multilevel Data: The Advantages of Multilevel SEM. Struct Equ Model Multidiscip J. 2011;18: 161–182. doi:10.1080/10705511.2011.557329

9. Westfall J, Yarkoni T. Statistically Controlling for Confounding Constructs Is Harder than You Think. Tran US, editor. PLOS ONE. 2016;11: e0152719. doi:10.1371/journal.pone.0152719

10. Muthén LK, Muthén BO. Mplus User’s Guide. Eighth Edition. Los Angeles, CA, USA: Muthén & Muthén; 1998.

11. Soto CJ, John OP. The next Big Five Inventory (BFI-2): Developing and assessing a hierarchical model with 15 facets to enhance bandwidth, fidelity, and predictive power. J Pers Soc Psychol. 2017;113: 117–143. doi:10.1037/pspp0000096

12. Michalak J, Heidenreich T, Ströhle G, Nachtigall C. Die deutsche Version der Mindful Attention and Awareness Scale (MAAS) Psychometrische Befunde zu einem Achtsamkeitsfragebogen. Z Für Klin Psychol Psychother. 2008;37: 200–208. doi:10.1026/1616-3443.37.3.200

13. Danner D, Rammstedt B, Bluemke M, Lechner C, Berres S, Knopf T, et al. Das Big Five Inventar 2: Validierung eines Persönlichkeitsinventars zur Erfassung von 5 Persönlichkeitsdomänen und 15 Facetten. Diagnostica. 2019;65: 121–132. doi:10.1026/0012-1924/a000218

14. Bainbridge TF, Ludeke SG, Smillie LD. Evaluating the Big Five as an organizing framework for commonly used psychological trait scales. J Pers Soc Psychol. 2022;122: 749–777. doi:10.1037/pspp0000395

1. When agreeableness is included, the effects of IER on sympathy and supportiveness are non-significant. However, when agreeableness is entered as an alternative mediator to sympathy, the indirect effect of IER on supportiveness remains significant. This further justifies not including it in the main analysis. [↑](#footnote-ref-1)
